# Combining dense and sparse labeling in optical DNA mapping

**DOI:** 10.1371/journal.pone.0260489

**Published:** 2021-11-29

**Authors:** Erik Torstensson, Gaurav Goyal, Anna Johnning, Fredrik Westerlund, Tobias Ambjörnsson

**Affiliations:** 1 Department of Astronomy and Theoretical Physics, Lund University, Lund, Sweden; 2 Department of Biology and Biological Engineering, Chalmers University of Technology, Gothenburg, Sweden; 3 Department of Mathematical Sciences, Chalmers University of Technology and the University of Gothenburg, Gothenburg, Sweden; 4 Systems and Data Analysis, Fraunhofer-Chalmers Centre, Gothenburg, Sweden; 5 Centre for Antibiotic Resistance Research, CARe, University of Gothenburg, Gothenburg, Sweden; University of Helsinki: Helsingin Yliopisto, FINLAND

## Abstract

Optical DNA mapping (ODM) is based on fluorescent labeling, stretching and imaging of single DNA molecules to obtain sequence-specific fluorescence profiles, DNA barcodes. These barcodes can be mapped to theoretical counterparts obtained from DNA reference sequences, which in turn allow for DNA identification in complex samples and for detecting structural changes in individual DNA molecules. There are several types of DNA labeling schemes for ODM and for each labeling type one or several types of match scoring methods are used. By combining the information from multiple labeling schemes one can potentially improve mapping confidence; however, combining match scores from different labeling assays has not been implemented yet. In this study, we introduce two theoretical methods for dealing with analysis of DNA molecules with multiple label types. In our first method, we convert the alignment scores, given as output from the different assays, into p-values using carefully crafted null models. We then combine the p-values for different label types using standard methods to obtain a combined match score and an associated combined p-value. In the second method, we use a block bootstrap approach to check for the uniqueness of a match to a database for all barcodes matching with a combined p-value below a predefined threshold. For obtaining experimental dual-labeled DNA barcodes, we introduce a novel assay where we cut plasmid DNA molecules from bacteria with restriction enzymes and the cut sites serve as sequence-specific markers, which together with barcodes obtained using the established competitive binding labeling method, form a dual-labeled barcode. All experimental data in this study originates from this assay, but we point out that our theoretical framework can be used to combine data from all kinds of available optical DNA mapping assays. We test our multiple labeling frameworks on barcodes from two different plasmids and synthetically generated barcodes (combined competitive-binding- and nick-labeling). It is demonstrated that by simultaneously using the information from all label types, we can substantially increase the significance when we match experimental barcodes to a database consisting of theoretical barcodes for all sequenced plasmids.

## 1 Introduction

Optical DNA mapping (ODM) is a method for generating sequence-dependent fluorescence “fingerprints” (DNA barcodes) along single DNA molecules [1]. Before imaging, the DNA molecules are labeled and stretched using nanochannels or surface adsorption. In contrast to DNA sequencing, ODM does not provide nucleotide level resolution; however, the method offers valuable contextual information for hundreds of kilobases long DNA molecules and has been very effective in detecting large structural variations that are missed by sequencing [2].

The most common method for fluorescent labeling DNA for ODM is *sparse labeling*. [3] In this method, each label can be identified in the barcode. This includes restriction enzyme cut DNA fragmentation mapping [4], where DNA molecules are digested by restriction enzymes and each cut site serves as a sequence-specific mark; enzymatic nick-labeling [5–7], where a nicking enzyme is used to make single-stranded cuts and then a polymerase is used to incorporate fluorescently labeled nucleotides at the cut sites; and finally, direct labeling and staining (DLS), where a methyltransferase (with a modified co-factor) is used to attach a fluorescent label at its recognition site. The use of sparsely labeled DNA barcodes has resulted in two types of commercially available platforms: the OpGen Argus [8] and the BioNano Genomics Irys and Saphyr systems [9–11].

In contrast to sparse labeling, in *dense labeling*, the sequence-dependent DNA fingerprint is a continuous intensity signal along the stretched DNA. This includes DNA melt mapping [12, 13], competitive binding (CB) [14–17] and dense enzymatic labeling [18]. We pioneered the use of the CB strategy for ODM. The CB assay is based on one-pot mixing of DNA with the fluorescent dye YOYO-1 and the non-fluorescent molecule netropsin that has high specificity for AT-rich regions and competes with YOYO-1 for AT sites. The result is an emission intensity variation along the DNA that corresponds to the underlying sequence—a DNA barcode. We have demonstrated the use of CB-based ODM for plasmid mapping [19–22], bacterial species identification [15, 17] and mapping fragments of the human genome [16].

The experiments in this study involve plasmids. These extrachromosomal, circular DNA molecules are of interest since they often encode genes that make bacteria resistant to common antibiotics. We have in several previous studies demonstrated how CB-based ODM can be used to characterize a sample by the number of plasmids and their sizes [19]. This was in turn used to identify a nosocomial spread of bacteria, as well as plasmid conjugation during resistance outbreaks in hospitals [21–23]. By combining the ODM assay with Cas9 we have also been able to identify the gene that makes the bacteria resistant [20].

Although the different labeling methods are robust by themselves, some of them could potentially be combined to generate dual- or multiply-labeled DNA barcodes. This would in principle allow the complementary labeling channels to support mapping in the areas of the genome where the primary labeling fails to provide sufficient mapping confidence. Despite the potential advantages of multiple labeling, there are currently no general-purpose theoretical frameworks for combining the mapping information from different types of assays.

In this study, we introduce a theoretical approach for combining the mapping information from sparsely and densely labeled DNA barcodes. Our method is based on converting alignment scores from different label types to p-values. The p-values, in turn, are obtained by utilizing randomized barcodes and functional fits based on extreme-value statistics. The different p-values are then combined using standard methods. Our p-value method allows us to separate significant (better than “random”) from non-significant matches when matching to a reference database.

We also introduce a new resampling method to determine the uniqueness-of-match of the top-scoring barcode compared to other barcodes which also match significantly to the reference database. Our block bootstrap resampling method generalizes the method from Bouwens [24] to include both dense-, sparse- and multiply-labeled DNA barcodes.

The experimental dual-labeled DNA barcodes used in this study were obtained by combining CB-based densely-labeled barcodes [14] with sparsely-labeled barcodes acquired from an enzymatic cutting of plasmid DNA molecules. The plasmids were partially digested resulting in a single cut (when multiple cut sites were present). This allowed us to align the molecules to each other using the CB barcode and identify the cut positions which then serve as sequence-specific markers. Compared to the original enzymatic cutting approach by Schwartz et al. [4] (see also [25]), our method allows us to preserve long DNA molecules (which are essential for ODM in nanochannels) during partial enzymatic digestion and still place a sufficient number of sparse labels on the DNA for robust mapping.

## 2 Methods

This methods section is organized as follows: We first describe the plasmid barcode database used herein and how it was generated. We later match experimental barcodes and mimicked experiments (synthetic barcodes) to the barcodes in this database. We then describe our method for experimentally generating dually labeled DNA barcodes (CB-labeled and cut-labeled barcodes) for plasmid DNA molecules. We then show how we generate synthetic CB and nick-labeled DNA barcodes. We proceed by introducing our framework for combining alignment scores for different label types. Finally, we describe our block bootstrap approach for addressing the uniqueness-of-match problem when matching barcodes to a database.

Our software is publicly available as a MATLAB package “cdsodm”, see the Data availability statement.

### 2.1 Database of plasmid reference DNA barcodes

In this study, we use a database consisting of theoretical DNA barcodes obtained from plasmid DNA sequences. The database is used for testing the capabilities of our new methods for identifying plasmids using multiply-labeled barcodes. To generate the database, we use reference DNA sequences from a public repository as input. The generated database contains barcodes corresponding to three different labeling assays (see Introduction): cut-labeled barcodes (our new experimental method for generating sparse labels, see next section), nick-labeled barcodes (sparse labeling), and CB barcodes (dense labeling). For each reference sequence, there are *N*_*c*_ + *N*_*n*_ + 1 barcodes in the database, where *N*_*c*_ is the number of enzymes used in the cut-labeling assay and *N*_*n*_ is the number of label types used for nick-labeling. Below we describe the public sequence repository and how theoretical barcodes for each labeling type are generated.

The public repository used is the NCBI (National Center of Biotechnology Information) RefSeq database [26] of fully sequenced plasmids. At the time of access (2021–05-18), the database contained 28293 plasmid sequences. Whether a comparison between experiment and database results in a unique match or not will depend on the database—there might be reference plasmids that are near-identical on the sequence level. We therefore made a further refinement of the RefSeq database, where we kept plasmids with complete and verified genomes (prefix ‘NC’) and of lengths between 50 kbps to 800 kbps. This resulted in 1420 plasmids with lengths roughly exponentially distributed with a mean length of 156 kbps.

Cut-labeled theoretical DNA barcodes were generated using enzymatic cutting at sequence-specific positions. Using DNA sequences from the RefSeq repository, we first locate the restriction sites for the enzyme at hand in the DNA sequence. We then represent the sparsely-labeled DNA barcodes by a sum of Gaussians, with a standard deviation *σ*_cut_ = 1.3 pixels (estimate from the type of experiments described in Sec. 2.2), centered at each restriction site. More precisely, we denote by $x_{j}^{\left(n\right)}$ the *j*th determined location of the sequence-specific markers (dots) for enzyme *n*. We then convert the dot positions to a sparsely labeled experimental barcode, *A*_*n*_(*x*), using:

$$\begin{array}{c} A_{n}\left(x\right)=\sum_{j=1}^{J^{\left(n\right)}}\text{exp}(-\frac{{\left[x-x_{j}^{\left(n\right)}\right]}^{2}}{2\sigma_{j}^{2}})\;, \end{array}$$ (1)

with *σ*_*j*_ = *σ*_cut_. Above, *x* denotes positions along the DNA barcode and *J*^(*n*)^ is the number of restriction sites for enzyme *n*. Finally, we convert the barcode *A*_*n*_(*x*) from base-pairs to pixels we use a moving mean of 1 pixel (≈ 758 base-pairs).

Nick-labeled theoretical DNA barcodes were generated in the same way as cut-labeled barcodes, with the exception that *σ*_*j*_ was instead set equal to the estimated standard deviation of the experimental point spread function: *σ*_j_ = *σ*_psf_ = 300nm ≈ 1.88 pixels [15].

The densely labeled DNA barcodes, *U*(*x*), in this study were based on the competitive binding (CB) method [14]. Using the DNA sequences from the repository as input, we generate theoretical CB barcodes using a procedure described previously [15, 27].

### 2.2 Dual labeling experiments using cut labeling and competitive-binding labeling

**Fig 1:**
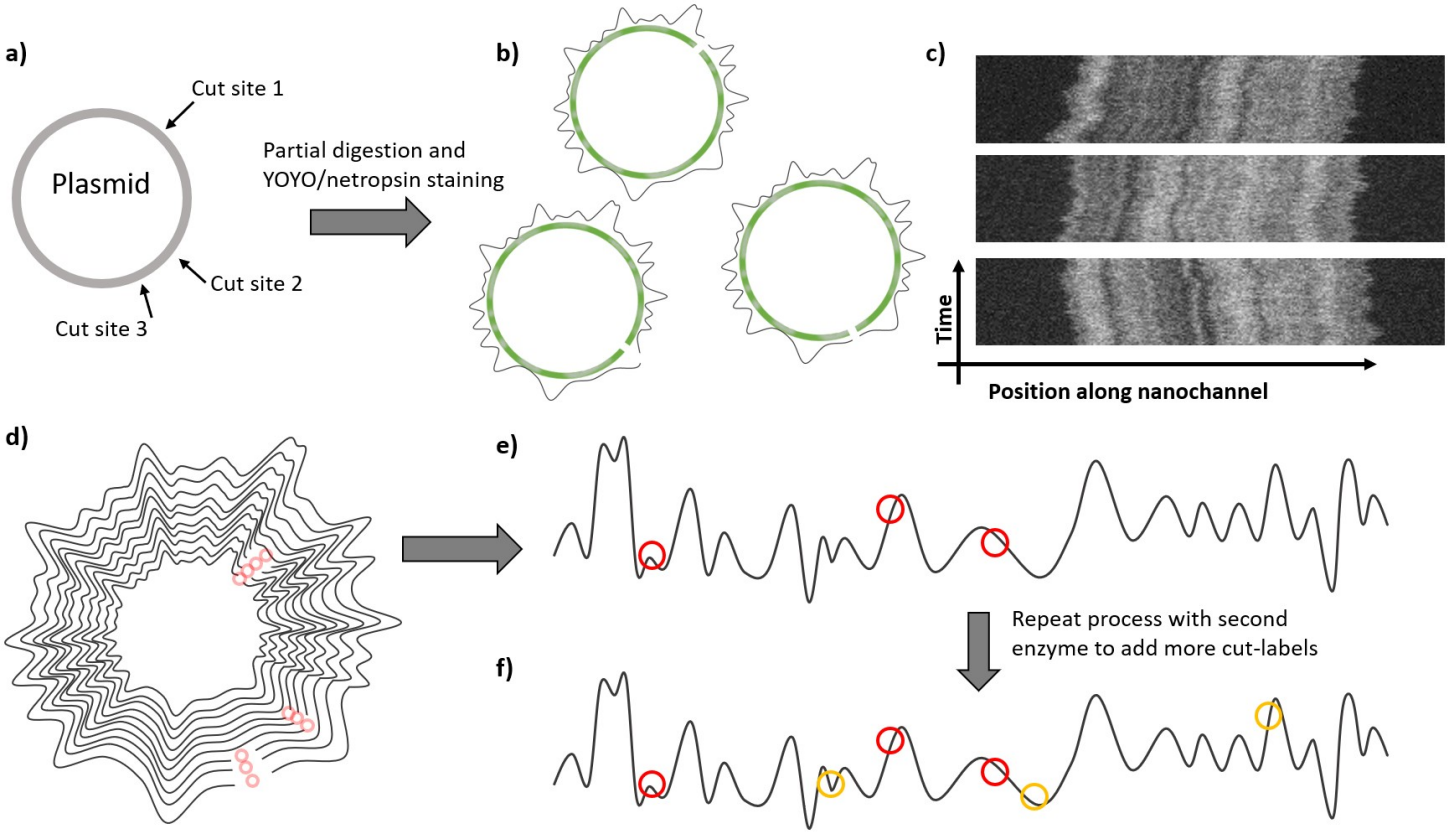
Generating dually labeled DNA barcodes using partial plasmid enzyme digestion and competitive binding labeling. a) Plasmid with three theoretical cut sites for a restriction enzyme. b) After the plasmid is partially digested and stained with YOYO/netropsin, we get three sub-populations with cuts at one of the three sites. Here green circles depict YOYO/netropsin stained plasmids and black circular curves around plasmids depict the fluorescent intensity profile. The discontinuity depicts enzyme cut on the plasmid. c) Shows representative experimental kymographs of three sub-populations when 130 kb plasmid was digested with the AscI enzyme. d) After imaging many molecules, their intensity profiles can be aligned to create a circular consensus plot. Here we see a circular consensus plot with three cut sites marked with red circles. e) A consensus intensity profile can then be obtained and cut-labels can be placed on it to generate a dually-labeled barcode. f) Potentially, the process can be repeated with another set of enzymes in order to add more cut-labels to the barcode.

All experiments in this study originate from two DNA samples containing plasmids of lengths 130 kbps and 220 kbps, respectively. S1 File provides details about plasmid sources and the isolation protocol. DNA molecules were labeled with both dense and sparse labels (CB- and cut-labeling). A schematic illustration of our experimental dual-labeling assay is found in Fig 1.

Sparse cut-labeling was achieved by enzymatic digestion of the plasmids. We wanted to partially digest the plasmids such that each molecule was cut only once even when more than one cut site for the enzyme was present. To this end, the 130 kbps plasmid was cut with AscI (New England Biolabs, 3 cut sites) or PmeI (New England Biolabs, 5 cut sites) and the enzyme concentration was titrated against the picomoles of cut sites (see S1 File). In this way, different molecules get cut at different cut sites and each single linear molecule carries the complete sequence. This strategy allowed us to align the molecules based on the dense CB labeling and use different cut sites as sparse sequence markers. However, partial digestion with a set of restriction enzymes requires knowledge of the number of cut sites and careful titration with enzymes to obtain full-length linear plasmids. Optimizing partial digestion for every enzyme set could slow down the process of data collection. Therefore, for the 220 kbps plasmid, we performed complete digestion by the restriction enzymes SgrDI (ThermoFisher Scientific, 4 cut sites), PacI (New England Biolabs, 4 cut sites) and PmeI (New England Biolabs, 3 cut sites). The restriction enzymes used are listed in Table 1.

**Table 1:** Restriction enzymes used in this study. The names and recognition sequences (restriction sites) of the restriction enzymes that were used to produce experimental dually labeled DNA barcodes. Note that the complement recognition sequences of each listed recognition sequences is a palindromic and that these enzymes cleave both strands of the DNA molecule. This means that when a plasmid reference sequence is searched for the recognition sequence, only one of the DNA strands has to be considered and only the 5’ to 3’ direction.

Enzyme	Recognition sequence
AscI	5’…GG-CGCGCC…3’
PmeI	5’…GTTT-AAAC…3’
PacI	5’…TTAAT-TAA…3’
SgrDI	5’…CG-TCGACG…3’

The densely labeled DNA barcodes were generated using the CB assay which has been described elsewhere [1, 14]. Briefly, digested plasmid samples were stained with YOYO-1 (YOYO, Invitrogen) and netropsin (Sigma Aldrich) using ratios basepair:YOYO:netropsin ∷ 10:1:300. λ-DNA (48502 bps, New England Biolabs) was included in the sample as an internal size reference. Staining using YOYO and netropsin creates a variable intensity profile along the DNA based on the relative distribution of AT/GC rich regions.

Labeled DNA molecules were stretched and imaged using nanochannels (microscopy setup and nanochannel imaging details are described in S1 File). For the partially digested 130 kbps plasmid, a mixed population of circular, partially digested (with a single cut, ∼130 kbps linear DNA) and fully digested plasmids were seen, see Fig 1. Only the linear ∼130 kbps long DNA molecules were used for further analysis. Further details are found in S1 File.

For the 220 kbps plasmid, all fragments were imaged (regardless of the level of digestion). The longest fragments were put together to form a consensus barcode using established methods [19]. Each of the shorter fragments was then positioned on this CB consensus barcode at an optimal position (the position which maximized the Pearson correlation coefficient). The edge positions of the fragments at these optimal positions served as cut labels.

Our post-processing procedures of the experimental cut position data are described in S1 File. The purpose of the post-processing is to locate the common sequence-specific cut positions with as high precision as possible using a set of aligned DNA barcodes, as well as removing false cuts. Once all cut positions have been located, these are converted into a sparsely-labeled barcode using Eq (1), where *σ*_*j*_ is the estimated standard deviation, *σ*_cut_, in the cut positions.

### 2.3 Generating synthetic barcodes with combined nick and competitive-binding labeling

Besides experimental barcodes, we also generate synthetic barcodes (mimicking experimental barcodes). These barcodes have the added value that the ground truth plasmid identity in the database is known. In this study, our particular use of synthetic barcodes is to investigate whether combining nick-labeled barcodes (the most common labeling strategy today) [28, 29] with CB barcodes can improve plasmid identification as compared to only using nick-labeling.

To generate synthetic nick-labeled barcodes, we proceed as follows: From the DNA sequence, we locate all specific sites for nick-labeling for a given plasmid DNA sequence. We then choose a probability for each site to be labeled and a rate of false-positive labeling [30]. We here choose to use the default parameters of the software OMTools [31]. The probability of a site being labeled is then 90%, and the rate of DNA having a false positive label is one per 100 kbps. The set of sites that are obtained this way are represented by a binary vector, where a ‘1’ corresponds to a nick-label at that position and a ‘0’ corresponds to no label. We then mimic the effect of the system’s point spread function by convolving the binary vector with a normal PDF (probability density function) with standard deviation *σ*_psf_ (standard deviation of the point spread function). Finally, we mix this nick-labeled barcode with a noise profile (the same type as described for densely labeled DNA barcodes below) at proportions *α* and (1 − *α*), where *α* = 0.025.

For generating synthetic densely labeled (CB) DNA barcodes, we start with the theoretical barcodes as calculated using the DNA sequence as input (see Sec 2.1). Background noise and shot noise [32] present in the experiment is then modeled as correlated normally distributed random numbers. To generate such numbers, we first generate a noise profile by drawing random numbers from a normal distribution with a mean equal to the mean intensity of the theoretical barcode and standard deviation 1 for each pixel (i.e., we draw random numbers from N(μ,1), where *μ* is the mean intensity of the theoretical barcode). We then mimic the effect of the system’s point spread function by convolving this noise profile with a normal PDF with standard deviation *σ*_psf_ (standard deviation of the point spread function). Finally, we mix the convolved noise profile with the theoretical barcode at proportions *α* and (1 − *α*), where *α* = 0.025. Through these steps, we generate a synthetic CB barcode. We validate our procedure for generating synthetic CB barcodes in S1 File, where we show that with the procedure above we get optimal alignment scores in agreement with experiments.

### 2.4 Combining alignment scores for different label types

In this section we show how to calculate alignment scores for densely- and sparsely-labeled DNA barcodes, by comparing experimental or synthetic barcodes to theoretical counterparts. Since plasmids are circular, we calculate such alignment scores for all possible circular shifts (and the two barcode orientations) of the barcodes being compared. We then convert optimal alignment scores (the maximum alignment score over all shifts and orientations) to p-values for each label type, and show how to merge these p-values into a single combined match score, *Z*, and an associated combined p-value.

#### 2.4.1 Alignment and match scores

Our new choice of alignment score, *D*, for sparsely labeled barcodes is a correlation coefficient that can be summed and normalized when using multiple sparsely-labeled barcodes in conjunction:

$$\begin{array}{c} D\left(A,B\right)=\frac{\sum_{n=1}^{N}[\sum_{x=1}^{L}A_{n}\left(x\right)B_{n}\left(x\right)]}{{\left(\sum_{n=1}^{N}[\sum_{x=1}^{L}{\left|A_{n}\left(x\right)\right|}^{2}]\right)}^{1/2}{\left(\sum_{n=1}^{N}[\sum_{x=1}^{L}{\left|B_{n}\left(x\right)\right|}^{2}]\right)}^{1/2}} \end{array}$$ (2)

Above, *A*_*n*_(*x*) (*B*_*n*_(*x*)) is the experimental (theoretical) sparsely-labeled barcode for (nicking or digestion) enzyme *n*, where *N* is the total number of enzymes. Note that the values of *D* are within the range [0, 1] (which follows from the Cauchy–Schwartz inequality). The score *D* is calculated for every possible orientation and circular shift of two barcodes, and the best overall score is denoted as $\hat{D}\left(A,B\right)$. The score $\hat{D}$ will henceforth be referred to as the *optimal alignment score for sparsely-labeled barcodes*.

A few comments regarding Eq 2 are in order. In previous studies, either a summed square difference between distances of dot locations on the experiment and theory, or, a Pearson correlation coefficient (PCC) was used [18, 33]. Our choice of score is similar to the PCC, but we do not subtract the means of the barcodes. In effect, our choice makes sure that non-matching regions in the barcodes provide no contribution to the overall score. In S1 Fig we compare our new sparse-labeling alignment score to the PCC, and find that in a large majority of cases the PCC and *D* yield the same best plasmid match in the database. The main reason we here use *D* instead of the PCC is that *D*, for our choice of null model (see Sec 2.4.2), has a distribution that is accessible in analytic form.

For densely labeled barcodes our choice of alignment score is the PCC as in previous studies [15, 21, 27]. In brief, for two densely labeled barcodes *U* and *V* of length *L* (number of pixels), the PCC is calculated using:

$$\begin{array}{c} C\left(U,V\right)=\frac{1}{L-1}\sum_{x=1}^{L}\frac{\left(U\left(x\right)-\mu_{U}\right)\left(V\left(x\right)-\mu_{V}\right)}{\sigma_{U}\sigma_{V}}\; \end{array}$$ (3)

Here, *μ*_*U*_, *μ*_*V*_ are the mean of the two barcodes, and *σ*_*U*_ and *σ*_*V*_ are associated standard deviations. The value of *C*(*U*, *V*) is within the range [−1, 1] where 1 is a perfect match. When the PCC has been calculated for every possible orientation and circular shift of two barcodes, the best overall score is denoted as $\hat{C}\left(U,V\right)$. The score $\hat{C}$ will henceforth also be referred to as the *optimal alignment score for densely labeled barcodes*.

Our two choices of scores above encompass all possible DNA barcodes (see Introduction).

#### 2.4.2 Null models and p-values

We here describe our procedures for converting the observed optimal alignment scores from the previous sections to p-values. The p-value measures how good an observed optimal alignment score is compared to that of a “random” match. More precisely, the p-value is computed using a null model which is represented by *M* randomized DNA barcodes. By comparing the experimental barcode at hand to all *M* barcodes, a null-model optimal-alignment-score histogram is obtained. By subsequently fitting a PDF to this histogram, the p-value is obtained as the area under the curve for scores above the observed optimal alignment score. We use *M* = 1000 throughout this study.

For sparsely labeled barcodes, we here introduce a new method for generating randomized sparsely-labeled barcodes. Our procedure to generate *M* randomized sparsely-labeled barcodes is: Draw with replacement 30 kbps long regions from the selected reference plasmids in the sequence repository. For each region identify the positions for the restriction sites (“dot positions”). This is repeated until a long dot-map has been produced that is *M* times the mean base-pair length of selected reference plasmids. Then draw *M* regions of length *L* with replacement from the randomized long dot-map. The resulting *M* dot-maps of length *L* are then turned into dot-barcodes using Eq (1). This procedure is the same for barcodes obtained using cut labeling and nick labeling. Examples of randomized dot-barcodes are found in S2 Fig.

**Fig 2:**
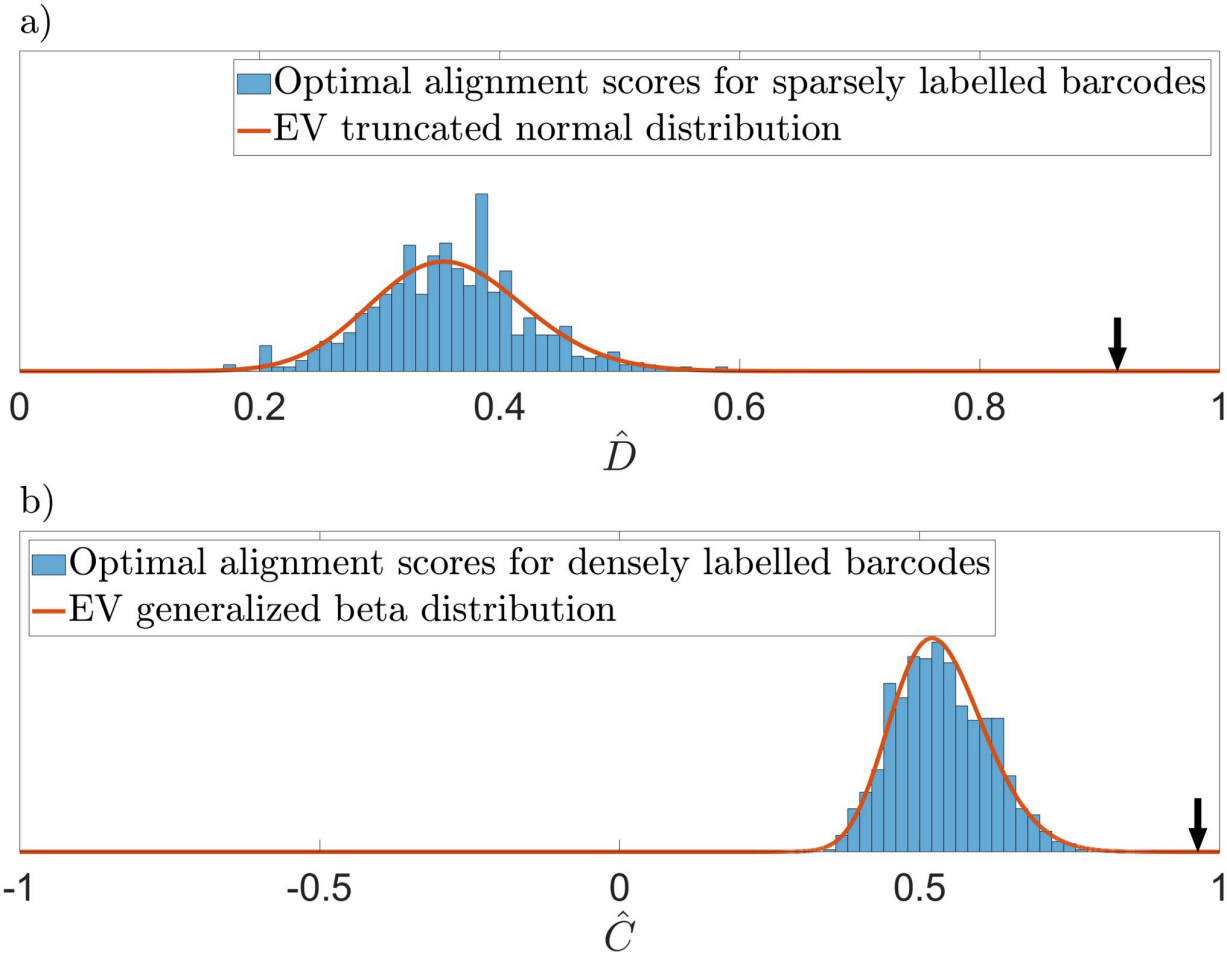
Optimal alignment score histograms obtained by matching to randomized densely- and sparsely-labeled barcodes. In order to generate a null model optimal alignment score histogram we first calculate many randomized barcodes, see S2 Fig for sparsely labeled barcodes and S3 Fig for densely labeled examples. We then calculate the alignment scores between the experimental barcode at hand and these randomized barcodes for every possible orientation and circular shift. Selecting the best score (optimal alignment) gives a score distribution that can be fitted to an extreme value distribution. (a) Optimal alignment score distribution for sparsely labeled barcodes. (b) Optimal alignment score distribution for densely labeled barcodes. Using these fitted distribution, we then convert any observed optimal alignment score to a match score *Z* and a p-value, as described in sec 2.4.2. The arrows in the two panels indicate the observed optimal alignment scores for the cases shown in Fig 4. Since these optimal alignment scores are far out in the tail of the distribution (p-values = 2 ⋅ 10^−10^ and 7 ⋅ 10^−12^, respectively), the optimal alignment scores in that figure are deemed to be significant. S4 Fig gives the distributions for all alignment scores (not just the best).

Once the *M* randomized dot-barcodes have been generated, we then match the experimental barcode at hand to all *M* randomized barcodes, and thereby generate a null-model optimal-alignment-score histogram. This histogram is fitted (using the maximum likelihood method) to the following extreme-value PDF (see S1 File for details):

$$\begin{array}{c} N_{\text{PDF}}^{\text{EV}}\left(x|\mu ,\sigma ,\lambda \right)=\lambda {\left(N_{\text{CDF}}^{\text{EV}}\left(x|\mu ,\sigma ,\lambda \right)\right)}^{\frac{\lambda -1}{\lambda }}\frac{1}{\sigma }\frac{N_{\text{PDF}}\left(\left(x-\mu \right)/\sigma \right)}{N_{\text{CDF}}\left(\left(1-\mu \right)/\sigma \right)-N_{\text{CDF}}\left(-\mu /\sigma \right)}\;, \end{array}$$ (4)

with three fit parameters: *μ*, *σ*, and λ. Also, $N_{\text{CDF}}\left(y\right)=(1+\text{erf}(y/\sqrt{2}))/2$ and $N_{\text{PDF}}\left(y\right)=\text{exp}(-y^{2}/2)/\sqrt{2\pi }$, where erf (*z*) is the Gauss error function and $N_{\text{CDF}}^{\text{EV}}\left(x|\mu ,\sigma ,\lambda \right)$ is given below. An example of an optimal-alignment-score histogram, with the associated fit, is found in Fig 2a).

Once the fit parameters have been obtained, we convert the observed optimal alignment score $\hat{D}$ to a p-value using:

$$\begin{array}{c} P_{\text{sparse}}=1-N_{\text{CDF}}^{\text{EV}}\left(\hat{D}|\mu ,\sigma ,\lambda \right), \end{array}$$ (5)

where we introduced the cumulative distribution function (CDF),

$$\begin{array}{c} N_{\text{CDF}}^{\text{EV}}\left(x|\mu ,\sigma ,\lambda \right)={\left(\frac{N_{\text{CDF}}\left(\left(x-\mu \right)/\sigma \right)-N_{\text{CDF}}\left(-\mu /\sigma \right)}{N_{\text{CDF}}\left(\left(1-\mu \right)/\sigma \right)-N_{\text{CDF}}\left(-\mu /\sigma \right)}\right)}^{\lambda }\;, \end{array}$$ (6)

associated with the PDF in Eq (4). Eq (5) is simply stating that the p-value is obtained as the area under the curve for scores above the observed optimal alignment score.

For densely-labeled DNA barcodes, the procedure for generating a null model (represented by randomized barcodes) and for calculating a p-values *P*_dense_, for competitive binding barcodes is described in [27]. In brief, randomized barcodes are generated using a phase randomization procedure. Examples of randomized barcodes matched to an experimental barcode at their optimal position are found in S3 Fig. The associated optimal-alignment-score histogram is found in Fig 2b).

For the other two types of dense labeling, DNA melt mapping and dense enzymatic labeling, the phase randomization procedure and functional fit procedure from [27] can be applied as is with the sole modification that the theoretical barcodes used as input in the phase randomization procedure must be replaced by their corresponding analogues.

#### 2.4.3 Combining p-value and generating a combined match score, *Z*

Unlike the individual optimal alignment scores, individual p-values are straightforward to combine into an overall p-value. To that end, we here use Stouffer’s method [34] which involves calculating a *Z*-score from a set of p-values, *p*_*i*_ (*i* = 1, …, *n*_*scores*_), according to:

$$\begin{array}{c} Z=-\frac{\sum_{i=1}^{n_{scores}}\eta_{i}\cdot N^{-1}\left(P_{i}|0,1\right)}{\sqrt{\sum_{i}\eta_{i}^{2}}}\;, \end{array}$$ (7)

where *n*_*scores*_ is the number of scores (number of p-values) being combined, $N^{-1}\left(P|\mu ,\sigma \right)$ is the inverse normal cumulative distribution function, and *η*_*i*_ are weight parameters (see below). By introducing a minus sign as a prefactor in Eq (7) we have that small p-values correspond to large positive *Z* values. We use the *Z*-score as defined in Eq (7) as our combined alignment score, and the best Z-score is referred to as our combined *match score* used when comparing two dually (or multiply) labeled barcodes. Note that we can also use Eq (7) to turn a single p-value (*n*_*scores*_ = 1) into a single match score.

By construction [34], the combined match score can be turned into a *combined p-value* using the normal cumulative distribution function according to:

$$\begin{array}{c} P_{\text{combined}}=1-N_{\text{CDF}}\left(Z\right)=\frac{1}{2}(1-\text{erf}(\frac{Z}{\sqrt{2}}))\;. \end{array}$$ (8)

By adjusting the weight parameters *η*_*i*_ the distribution of *Z* can be given a bias with respect to the individual p-values *p*_*i*_. In this study we, however, combine the two p-values *P*_dense_ and *P*_sparse_ using equal weights: *η*_dense_ = *η*_sparse_ = 1.

As demonstrated in Sec 2.4.2 and in this section, we now have means for calculating p-values (and associated match scores) for three cases: (i) sparsely-labeled barcodes matchings to the database, (ii) densely-labeled barcodes matchings and (iii) dually-labeled barcode matchings. A small p-value indicates a good match (as compared to a match against a randomized barcode). As in previous studies [21], we use a p-value threshold *p*_thresh_ = 0.01 to separate between significant and non-significant matches.

### 2.5 Uniqueness-of-match to a database and block bootstrap resampling

When matching an experimental barcode to our database of theoretical barcodes, there may be several barcode matches that are deemed significant (p-values smaller than *p*_thresh_). As an example scenario where this could happen, imagine two experimental barcodes: one is perfectly matching to theory (within experimental errors), whereas for the other barcode only, say, half the barcode is perfectly matching. Both of these barcode matches will be deemed significant in the sense that their matches to theory are better than to a randomized barcode. Yet, we would probably be inclined to say that the first barcode, the top match, is a “better” match compared to the second-best match, given the database at hand. How to deal with such cases?

In Bouwens et. al. [24] a resampling type approach for dealing with this type of problem was introduced. By “reshuffling” (resampling) the dot positions of the top matching theoretical dot-barcode, the authors were able to estimate the variance in match score for the top case. Based on this variance, one then tests whether the second-best match is within those fluctuations (non-unique top match) or not (a unique top match). Below, we extend the ideas from [24] by introducing a resampling method that is sufficiently general to include also densely- and multiply-labeled barcodes as well as can cope with experiment-to-experiment comparisons (the framework in [24] deals with experiments versus theory for sparsely-labeled barcodes, only).

In order to evaluate whether the top significant match is statistically better than other significant matches, we would ideally like to repeat the experiments many times. We would then simply count how many times a particular match is a top match, and if in a large majority of cases this particular match ends up at the top match, then we deem this match unique. In practice, this would typically be very time-consuming. Instead, a resampling method is presented here that serves as a poor man’s variant of performing repeated experiments.

**Fig 3:**
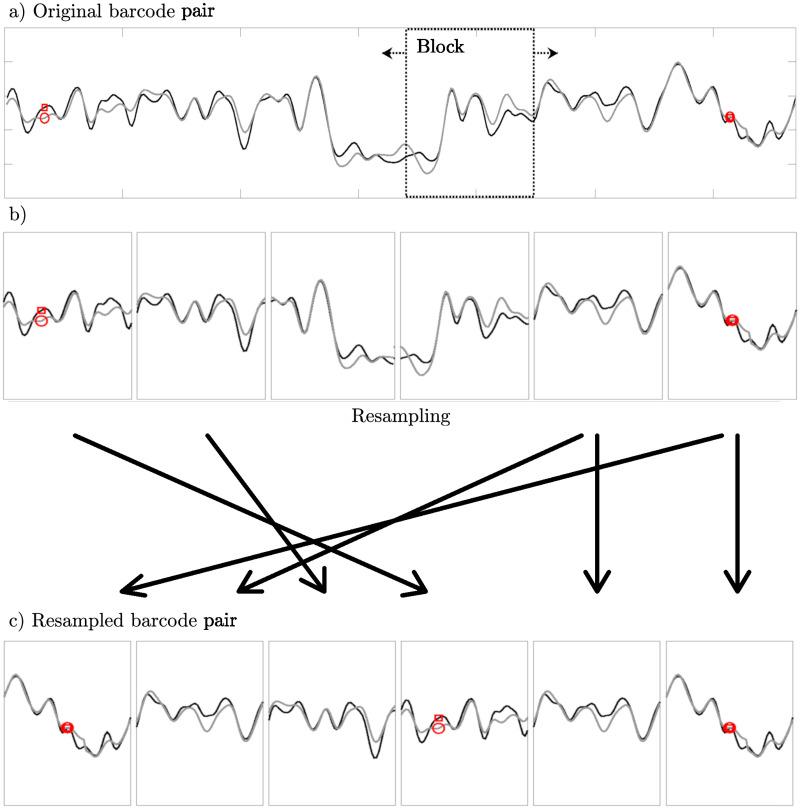
Resampling of a top-matching dually labeled barcode pair using the block-bootstrap method. The goal, as described in Sec 2.5, is to estimate the variance in the match-score of a top matching database plasmid. (a) The top-matching experimental barcode and the optimally aligned theoretical barcode are divided into blocks of equal size. (b) The blocks are sampled with replacement (see sec 2.5) and glued together into (c) a resampled barcode pair. The resampled barcode pair is used as if it were a variation of the original barcode and to calculate a new match-score. This can be repeated as many times as necessary to produce a set of resampled match scores. From this set, we calculate a sample estimate of the match-score variance for the top case. In the present study, we choose to generate 1000 resampled barcode pairs. Note that the blocks in the figure are larger than the ones used in practice for visual purposes.

In short, we consider the top matching barcode pair (experiment versus theory) at optimal alignment see Fig 3a). We then simultaneously divide the two barcodes into contiguous blocks, Fig 3b), larger than the correlation length (see below). In the next step we resample these blocks, with replacement, using the block-bootstrap procedure [35], as shown in Fig 3c). We then calculate a match-score for the resampled barcode pair. By repeating this procedure many times, a variance is estimated for the top match-score *Z*_dual_ (Eq (7)). Ideally, this variance is close to the variance in the top match-score that would appear due to experimental-to-experiment variations and the resampling procedure is then a substitute for repeating the experiment many times.

In detail, our procedure is:

1. Barcodes of plasmids with statistically significant match-scores (*P*_combined_ ≤ *p*_thresh_ = 0.01) are identified. The top barcode pair (a theoretical barcode and its experimental equivalent) is divided into equally sized blocks of width *W* (we here use *W* = 15pixels ≈ 8 *σ*_*PSF*_).
2. Blocks from the top densely- and sparsely labeled barcode pair are simultaneously randomly sampled with replacement (we pick blocks at random, and allow for the same block to be picked several times) and then “glued” together into resampled barcode pairs. This process is repeated, generating in total *M* resampled barcode pairs (in this study, *M* = 1000).
3. The resampled barcode pairs are compared to each other and a set of match-scores are obtained. From this set, we calculate the sample variance, *σ*_*Z*_.
4. The uniqueness of match: A database plasmid is said to have passed the resampling test if its match-score is within three *σ*_*Z*_ of *Z*_dual_ for the top-matching barcode pair.

Table 2 provides a fictive example of our resampling procedure for the case of five plasmids.

**Table 2:** Fictional example of how five plasmids sorted by match-score *Z* would be separated into the two levels of precision: ‘Significant match-score’ and ‘passed resampling test’. In this table five imaginary plasmids have been sorted by match-score *Z*. The top 4 plasmids are found to have *P*_dual_ ≤ 0.01, i.e., these plasmids are deemed to have statistically significant match scores. However, only the top 2 plasmids are considered to pass the resampling test, where a 3-*σ*_*Z*_ rule is used to exclude any plasmid where the difference in match-score, compared to the top case, is deemed to be too large.

Plasmid	*Z*_dual_	*σ*_*Z*_	*P*_dual_	Significant match-score	Passed re-sampling test
1	7	0.8	1.3 ⋅ 10^−12^	True	True
2	6	—	9.9 ⋅ 10^−10^	True	True
3	4	—	3.2 ⋅ 10^−5^	True	False
4	3	—	1.4 ⋅ 10^−3^	True	False
5	2	—	2.3 ⋅ 10^−2^	False	False

A few comments regarding our method are in place. In step 1 above, the block width was chosen such that it is just small enough to divide barcodes into two blocks for the shortest database plasmids that can typically be matched (∼20 kbps). The block width is also chosen to be longer than the length over which barcode intensity values are correlated (these correlations are mainly due to the point spread function of the imaging system). In step 2, note that both experimental barcodes and matching theoretical barcodes are simultaneously divided into blocks in order to keep track of the optimal alignment for each block. The method described here is inspired by error estimation of correlated data [36, 37].

## 3 Results

In this section, we first present the results of matching dual-labeling experiments (cut- and CB-labeling) to all theoretical barcodes in our plasmid barcode database (Sec 3.1). We show that by using dual labeling we increase the significance of matches as compared to using single labels. We then proceed to consider nick- and CB-labeled synthetic barcodes (Sec 3.2).

To speed up computations, whenever an experimental plasmid barcode or synthetic barcode is compared to the reference barcodes in the database, we make a pre-selection of reference barcodes within ±10% of the estimated experimental length.

### 3.1 Cut- and CB-labeling experiments versus theory

**Fig 4:**
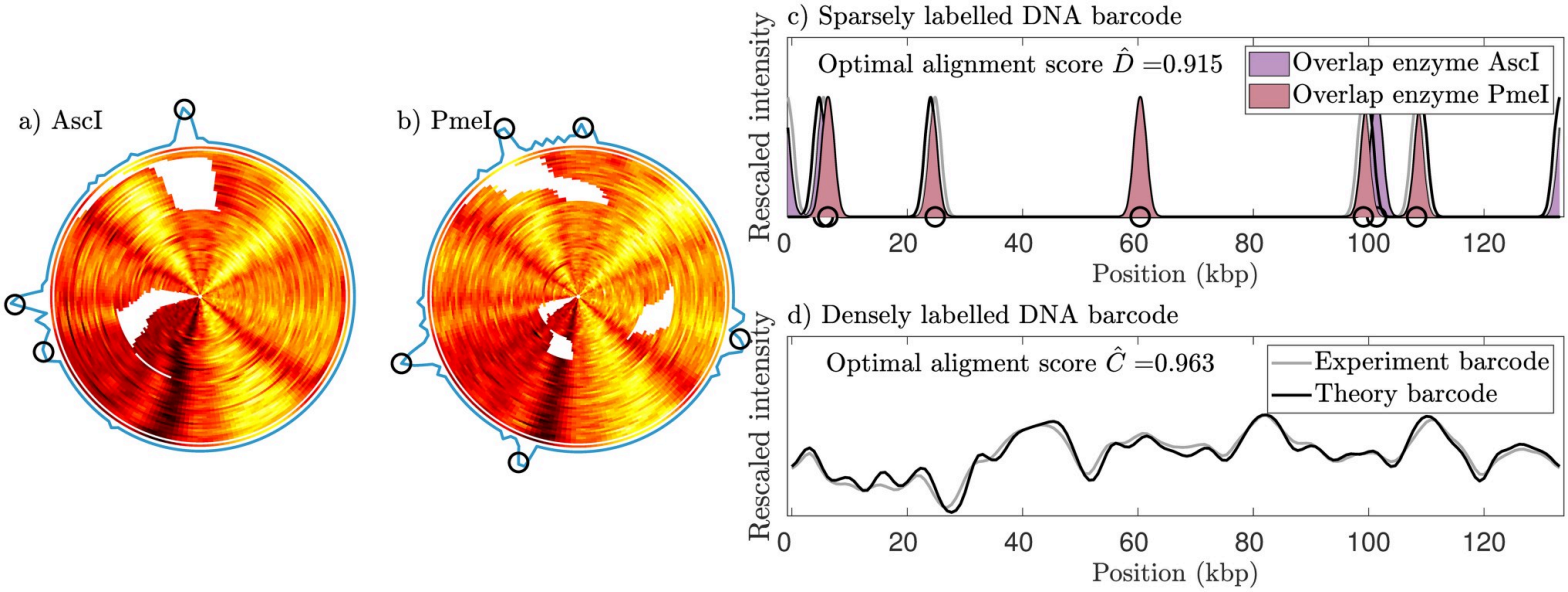
Experimental dually-labeled barcodes for the 130 kbps plasmid. The 130 kbps plasmids were digested sub-optimally (one cut per molecule) using two different enzymes [(a) AscI and (b) PmeI]. DNA barcodes for these digested plasmids were obtained through dense labeling (competitive binding assay). The experimental competitive binding barcodes were aligned into a consensus barcode as illustrated in the concentric plots above. The locations of cuts for each barcode are shown as the white regions. The determined locations of significant clusters of cuts (see Methods) are shown by the black circles (the sparse labels). The enzyme AscI produced three cuts while the enzyme PmeI produced five cuts. (c) Two theoretical cut-labeled barcodes have been aligned to the corresponding experimental cut-labeled barcodes in the orientation and circular shift where the alignment score, Eq (2), is maximized. The regions of overlap between experimental and theoretical barcodes corresponding to the same enzyme have been highlighted. (d) Densely labeled experimental consensus barcode aligned to the corresponding theoretical barcode. The alignment is at the orientation and circular shift where its alignment score, Eq (3), is maximized. The intensity values have been re-scaled such that the intensity values of each barcode have the same mean and standard deviation as the other.

The partially digested 130 kbps plasmid is used for the first demonstration. The experiment involves two data sets of the 130 kbps plasmid where the DNA sample for each data set was partially digested by the restriction enzymes AscI and PmeI, respectively. After post-processing, see S1 File, a consensus CB barcode and a set of significant cut locations (black circles) were obtained, as seen in Fig 4, panels a) and b). Fig 4, panel c), shows the experimental consensus CB barcode that has been aligned to its theoretical counterpart in the orientation and circular shift that maximizes its alignment score (see 2.4.1 and Eq 3). The optimal alignment score, $\hat{C}$, is also given in the figure. The significant cut locations identified in the barcodes were then turned into two cut-labeled barcodes and in turn compared to the theoretical barcodes, using Eq (2), as seen in Fig 4, panel (d). The optimal alignment score, $\hat{D}$, is also given in the figure.

**Fig 5:**
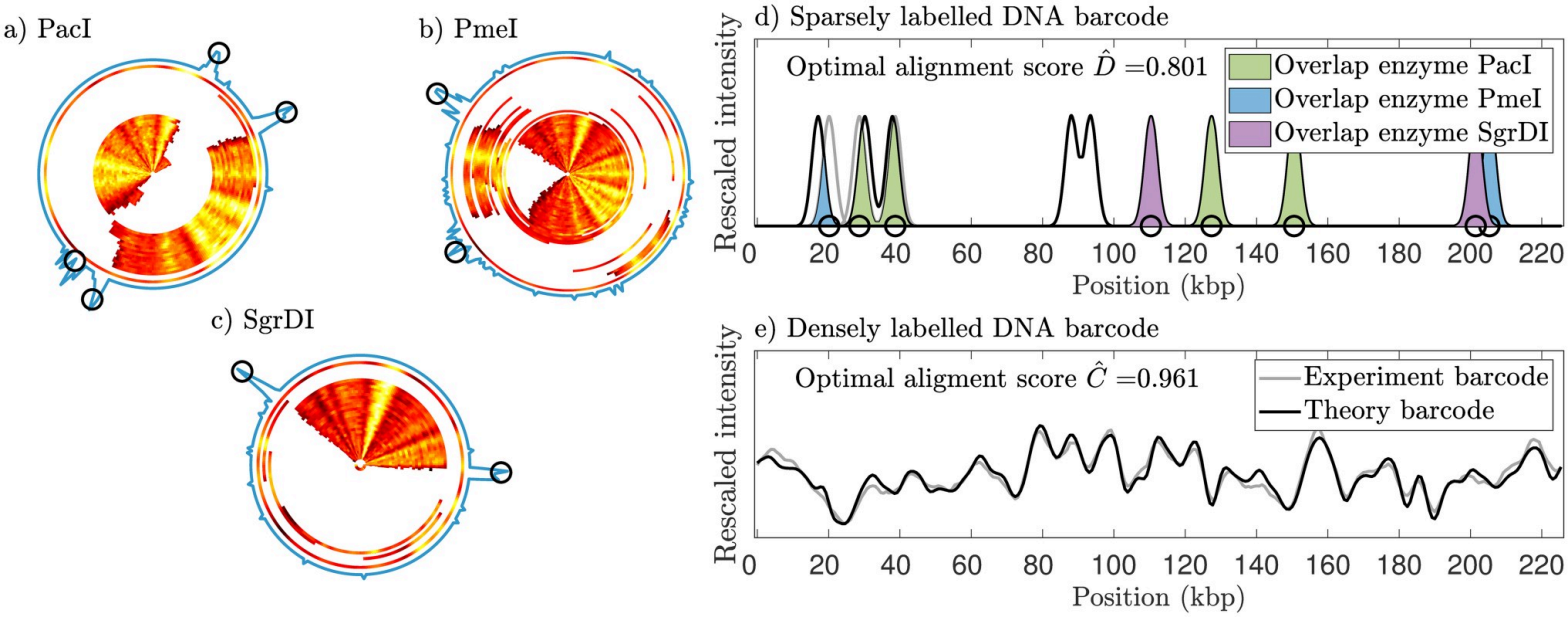
Experimental dually labeled barcodes for the 220 kbps plasmid. Three data sets containing fragments of a 220 kbps plasmid were obtained using competitive binding barcoding and sparsely cut-labeled barcodes obtained through full digestion using the restriction enzymes (a) PacI, (b) PmeI, and (c) SgrDI. The experimental competitive binding barcodes were aligned into a consensus barcode. The locations of cuts for each barcode are shown as white regions and the determined locations of significant clusters of cuts (see Methods) are shown by the black circles. (d) Three theoretical cut-labeled barcodes have been aligned to the corresponding experimental cut-labeled barcodes in the orientation and circular shift where the alignment score, Eq (2), is maximized. Enzyme PacI produced four cuts while enzyme PmeI and enzyme SgrDI produced two cuts each. The regions of overlap between experimental and theoretical barcodes corresponding to the same enzyme have been highlighted. (e) Densely labeled experimental consensus barcode aligned to the corresponding theoretical barcode. The alignment is at the orientation and circular shift where its alignment score, Eq (3), is maximized. The intensity values have been re-scaled such that the intensity values of each barcode have the same mean and standard deviation as the other.

As a second demonstration, a similar analysis was performed for the 220 kbps plasmid. For this plasmid, we demonstrate dual labeling with complete digestion of the plasmid with restriction enzymes PacI, PmeI and SgrDI. Since we did not expect full-length linear molecules, all the cut fragments were imaged. After post-processing, a consensus CB barcode, as well as three sets of significant cut locations, were obtained, as seen in Fig 5, panels a), b), and c). The consensus CB barcode and the three cut-labeled barcodes were then compared to their theoretical counterparts (see 2.4.1), as seen in Fig 5, panel d) and e). The optimal alignment scores, $\hat{C}$ and $\hat{D}$, are also given in the figure.

The comparison of experimental and theoretical sparse labels for the 220 kbps plasmid highlights the value of partial digestion. Since it is difficult to map small fragments in the nanofluidics setup, all theoretical cut sites for PmeI and SgrDI could not be captured in the experimental data when full-length fragments were not available (The theoretical digestion maps of the 220 kbps plasmid for PacI, PmeI and SgrDI are shown in S1 File). These results demonstrate that the performance of the assay when the plasmid is fully digested depends on the distance between the cut sites. All cut sites for PacI could be mapped; however, the cut sites for PmeI and SgrDI that were too close to one another could not be mapped. We here limit ODM to fragments larger than 30 kbps for practical purposes. However, as we demonstrated in [27], we can position CB barcodes as small as 12 kbps, provided that we use a p-value threshold to discard false positives. Although the complete plasmid digestion strategy does not always allow all cut sites to be mapped, it is much faster than the partial digestion approach as no digestion optimization is needed and prior knowledge of the number of cut sites is not required. We place eight cut labels on the 220 kbps plasmid (total theoretical cut labels for the three enzymes are eleven) but it is possible to set up more digestion reactions with other restriction enzymes to increase the label density.

This novel experimental assay relies heavily on the enzymes used for the plasmid restriction. One would ideally avoid enzymes that produce too few cuts, but one would also like to avoid having many closely-spaced cuts (within the spatial resolution limit). Therefore, for an unknown plasmid, it is not straightforward to pick a set of enzymes that are “suitable”. In the S1 File, we present an analysis that generalizes our findings and presents how to select an enzyme library that is expected to work well for a plasmid sample with unknown content.

**Fig 6:**
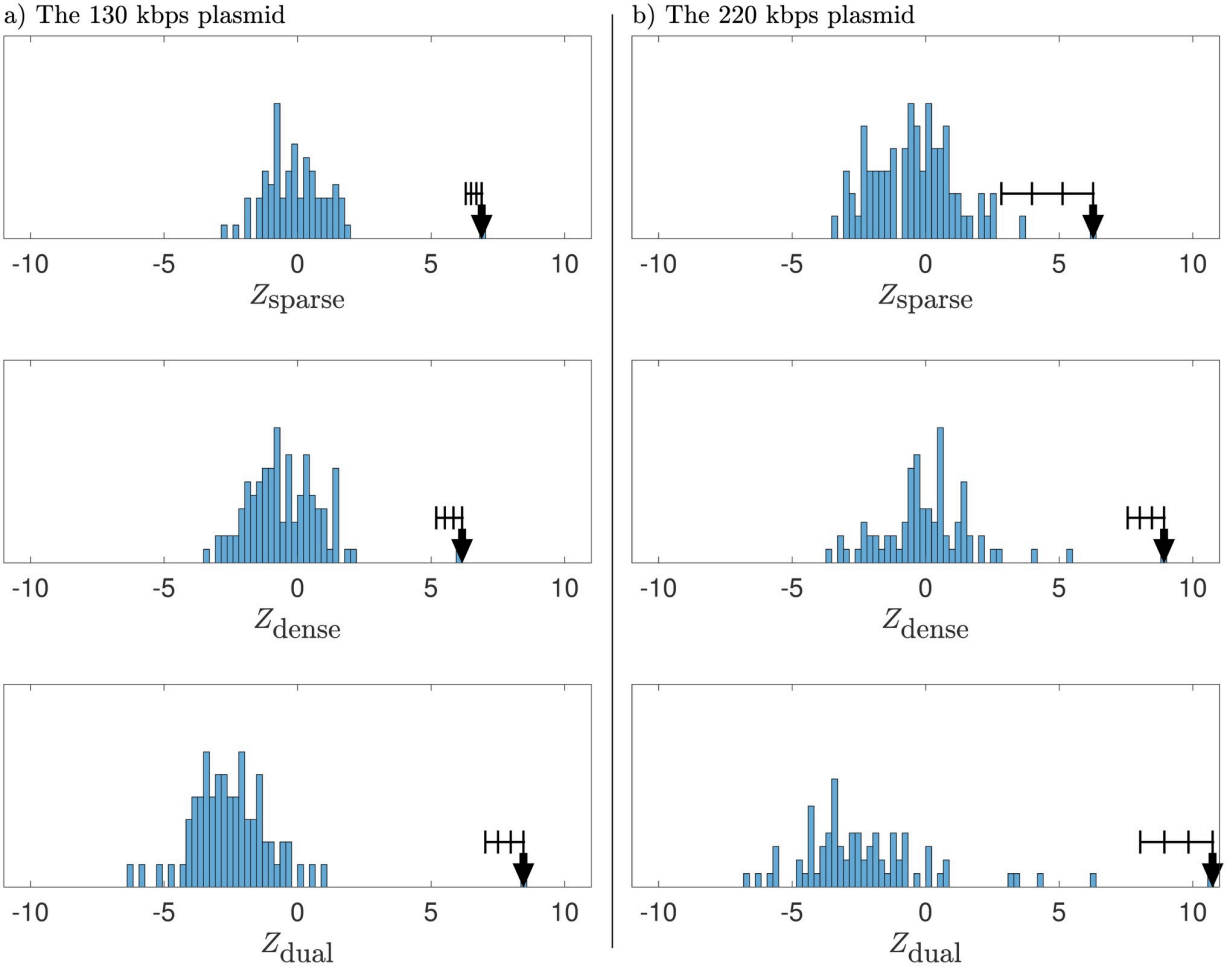
Match scores for the two plasmid barcodes against the plasmid barcode database. Histograms of match scores are obtained by comparing experimental dually labeled barcodes in Figs 4 and 5 against all theoretical barcodes in the plasmid barcode database. The histograms show match scores for each barcode type (top row = CB labeling, middle row = cut-labeling) as well as the combined match-score (bottom row). The arrows indicate the match scores at the optimal align position with respect to the combined match score. The error bars represent the uncertainty in the maximum match-scores as estimated by the bootstrap resampling procedure described in sec 2.5. For the 130 kbps plasmid, the p-values are *P*_sparse_ = 2.8 ⋅ 10^−12^, *P*_dense_ = 3.7 ⋅ 10^−10^, *P*_combined_ = 1.4 ⋅ 10^−17^. For the 220 kbps plasmid the p-values are *P*_sparse_ = 1.8 ⋅ 10^−10^, *P*_dense_ = 2.3 ⋅ 10^−19^, *P*_combined_ = 3.3 ⋅ 10^−27^. A quantitative measure of the uniqueness-of-match of the top case is how many, *X*, standard deviations (*σ*_*Z*_) there is between the *Z*-values for the top and second-best case. For the 130 kbps plasmid, we have *X*_sparse_ = 25.0, *X*_dense_ = 13.0, *P*_combined_ = 16.0. For the 220 kbps plasmid, we find *X*_sparse_ = 2.3, *X*_dense_ = 7.7, *X*_combined_ = 4.9.

Next, we tested our framework for combining the information from the two types of labeling. In Fig 6, we show histograms with the individual and combined match-scores *Z* for all database plasmids within the length threshold of the 130 kbps and the 220 kbps plasmids, respectively. For these two plasmids, we find that the experimental barcodes matched uniquely for both individual label types, suggesting that both dense and sparse labeling from our experiments is robust enough to generate unique matches for the two plasmids against the plasmid barcode database. However, the combined match score (see sec 2.4.3) was significantly larger than any of the two individual match scores. As a consequence, the combined p-value was significantly lowered (10^−17^) compared to the two individual p-values (10^−10^ and 10^−9^ for densely- and sparsely labeled barcodes, respectively).

### 3.2 Nick- and CB-labeled synthetic barcodes versus theory

**Fig 7:**
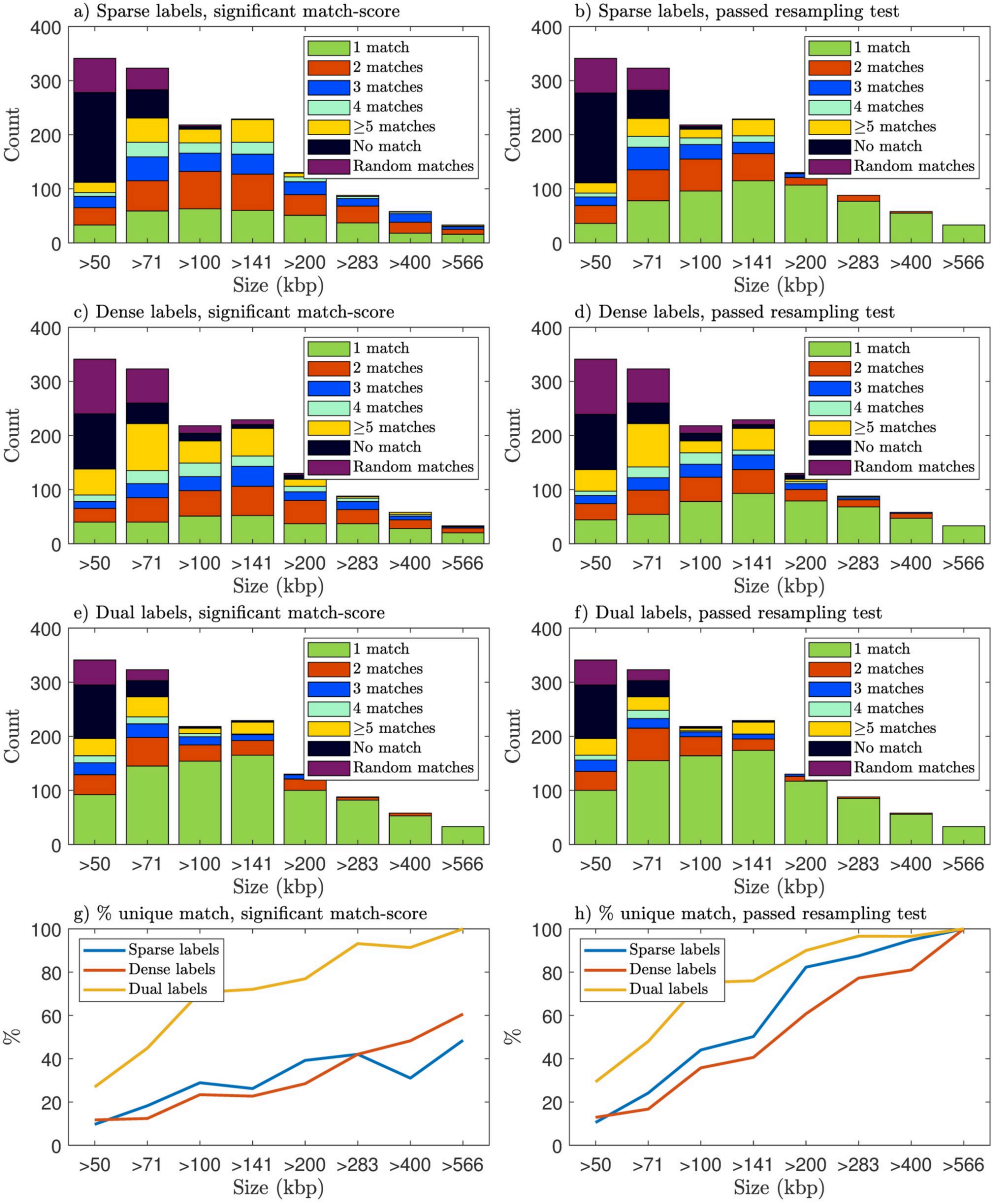
Number of matches when comparing all synthetic plasmid barcodes (nick- and CB-labeling) to the theoretical barcodes in the plasmid barcode database. For each plasmid in the database, the experiment for obtaining nick-labeled barcodes was emulated using the nicking enzyme BspQI. CB barcodes were obtained as described in Methods. The panels on the left show the number of matches with statistically significant match-scores, and the panels on the right show the number of best subset matches (passed the resampling test). The plasmids are sorted and binned by their lengths. ‘No match’ means that no database plasmid had a statistically significant match-score, and ‘Random matches’ means that matches were found, but none of them were the correct plasmid. A unique (and correct) match is highly preferred, so the larger the proportion of green in the bar is, the better is the matching performance to the database. Note, however, that whether we get a unique match will depend on the database at hand. This is the main reason that we restrict the database to complete and verified genomes, see Methods. The proportion of unique (and correct) matches is given above each stacked bar.

Next we investigate how barcodes obtained through nick-labeling could potentially be made more informative if combined with CB labeling. To that end, we mimic experiments by generating synthetic barcodes for all plasmids as described in Sec 2.3 in Methods. These synthetic barcodes, within the length threshold of ±10%, were then compared to the plasmid barcode database (which contains theoretical barcodes, see Sec 2.1 in Methods) using the matching procedure described in Methods (Sec 2.4). The number of plasmids matches at the two levels of precision, *statistically significant match-score* and *passing the resampling test*, were calculated. This number was calculated for three cases: (i) sparsely-labeled barcodes, (ii) densely-labeled barcodes, and (iii) dually-labeled barcodes. In Fig 7 we show stacked-bar-plots of the distribution of the number of matches to the plasmid database, sorted by plasmid length. The panels on the left show the number of matches with statistically significant match-scores, and the panels on the right show the number of best subset matches (plasmids that passed the resampling test) using the block-bootstrap resampling procedure. It is clear that single CB- or nick-labeling yields similar results when it comes to the fraction of matches. Importantly, we find that for all length ranges using dual labeling instead of nick-labeling improves, in a pronounced way, the matching to the database. Also, the longer the plasmid is, the better is the matching performance.

## 4 Summary and outlook

In this study, we investigated DNA barcodes with multiple label types. In particular, we introduced a theoretical method that allows for combining the alignment scores from different labeling schemes. We also put forward a novel method for dealing with the uniqueness-of-match problem when matching barcodes to a database using a block-bootstrap approach. To experimentally generate dually-labeled DNA barcodes, we introduced an experimental assay that uses competitive binding combined with restriction enzyme cutting. We tested out our frameworks by matching multiply-labeled experimental DNA barcodes, and synthetic barcodes, from plasmids to a database of theoretical barcodes for all sequenced plasmids. In general, we found that using multiply-labeled barcodes can significantly improve the number of matches to a barcode database as compared to using only a single label type.

The present study opens up for combining the results of several kinds of ODM assays for enhanced DNA analysis capabilities. The commercially available ODM systems, such as BioNano Genomics Saphyr, work with sparse labels (nick or DLE labeling chemistries); however, in the BioNano workflow the DNA backbone is still stained with YOYO-1 for the analysis pipeline to recognize the DNA molecules and localize dots on them. This DNA staining with YOYO-1 could be very easily extended to CB staining by including netropsin in the backbone staining step and converting the sparse labeled DNA into dual-labeled DNA. As demonstrated in this work, dual labeling can then be used to improve mapping accuracy. This kind of dual labeling could be especially useful for providing mapping support in the genomic regions where the sparse labeling is not sufficient (because of lower dots coverage), locally fails (epigenetic modification of labeling motif prevents label attachment, fluorophore bleaching, blinking etc), or when critical structural variations are present between two dots.

We hope the experimental demonstrations and the theoretical framework introduced in this work for combining sparse and dense labels will stimulate further efforts towards multi-labeled ODM.

## Supporting information

S1 Fig The PCC alignment score versus our new alignment score *D* for sparsely-labeled DNA barcodes. We ran a comparison of all synthetic sparsely-labeled DNA barcodes towards the plasmid database and identified the top plasmid (the plasmid with the largest alignment score). We find that in around 96% of the cases, the same plasmid ends up as the top case. In all of these cases is the optimal position identical for the two types of alignment scores.
(TIF)

S2 Fig Randomized dot barcodes. Two examples of randomized sparsely-labeled barcodes (cut-labeling) compared to an experimental barcode of the same length. Cut-labeled barcodes were obtained for the enzymes AscI and PmeI. By generating many such randomized barcodes and matching experiments to these, we get optimal alignment score distributions of the form in Fig 2a) in the main text.
(TIF)

S3 Fig Randomized densely-labeled DNA barcodes. Two examples of randomized densely-labeled barcodes (competitive binding) compared to an experimental barcode of the same length. By generating many such randomized barcodes and matching experiments to these, we get optimal alignment score distributions of the form in Fig 2b) in the main text.
(TIF)

S4 Fig Alignment score distributions for all positions. (a) When sparse-labeling alignment score, Eq (2) in the main text, is calculated between sufficiently long barcodes for every possible orientation and circular shift, the distribution is well described by a truncated normal distribution. (b) When the densely-labeling alignment score, Eq (3) in the main text, is calculated between sufficiently long barcodes for every possible orientation and circular shift, the distribution is centered around 0 and fitted by a functional form given in [27]. Compare these results to Fig 2 in the main text, which shows the associated optimal alignment score distribution for the best alignment.
(TIF)

S1 File Supporting methods. Here we provide further details about the experiments and theoretical methods.
(PDF)
