# Supplementary figures and images for: Combining dense and sparse labeling in optical DNA mapping
Source: PLoS One. 2021 Nov 29;16(11):e0260489. doi: 10.1371/journal.pone.0260489 (PMC8629184; doi:10.1371/journal.pone.0260489)

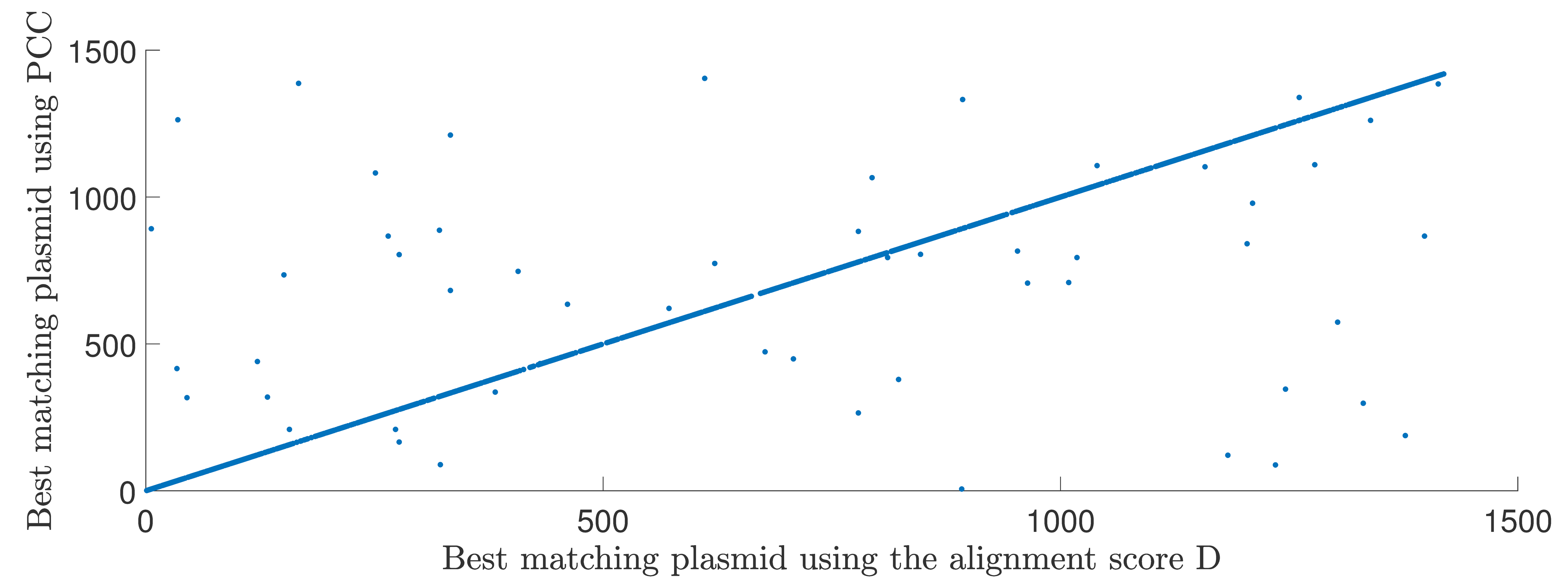

Supplement: S1 Fig — We ran a comparison of all synthetic sparsely-labeled DNA barcodes towards the plasmid database and identified the top plasmid (the plasmid with the largest alignment score). We find that in around 96% of the cases, the same plasmid ends up as the top case. In all of these cases is the optimal position identical for the two types of alignment scores. (TIF) [file pone.0260489.s001.tif]

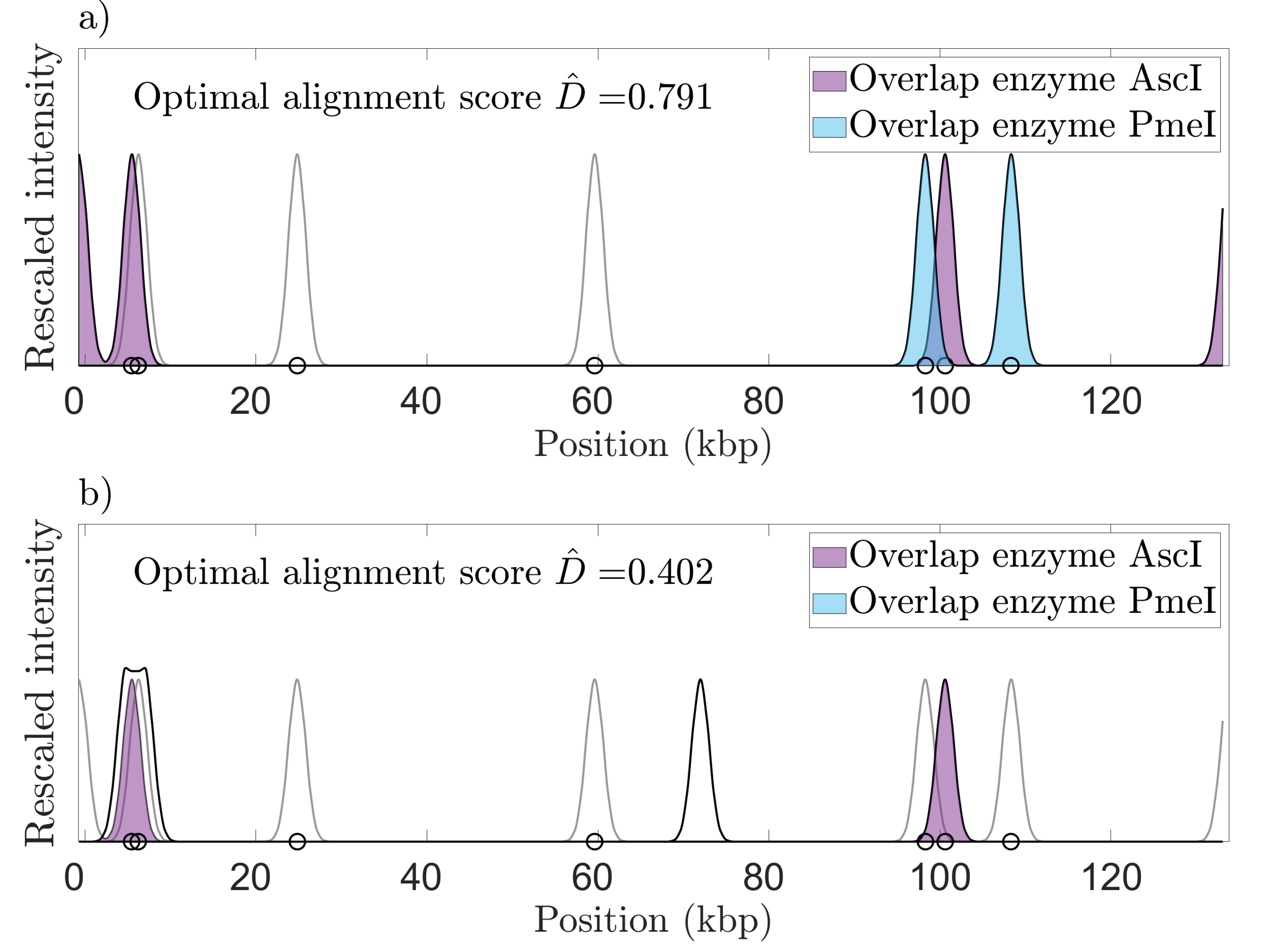

Supplement: S2 Fig — Two examples of randomized sparsely-labeled barcodes (cut-labeling) compared to an experimental barcode of the same length. Cut-labeled barcodes were obtained for the enzymes AscI and PmeI. By generating many such randomized barcodes and matching experiments to these, we get optimal alignment score distributions of the form in Fig 2a) in the main text. (TIF) [file pone.0260489.s002.tif]

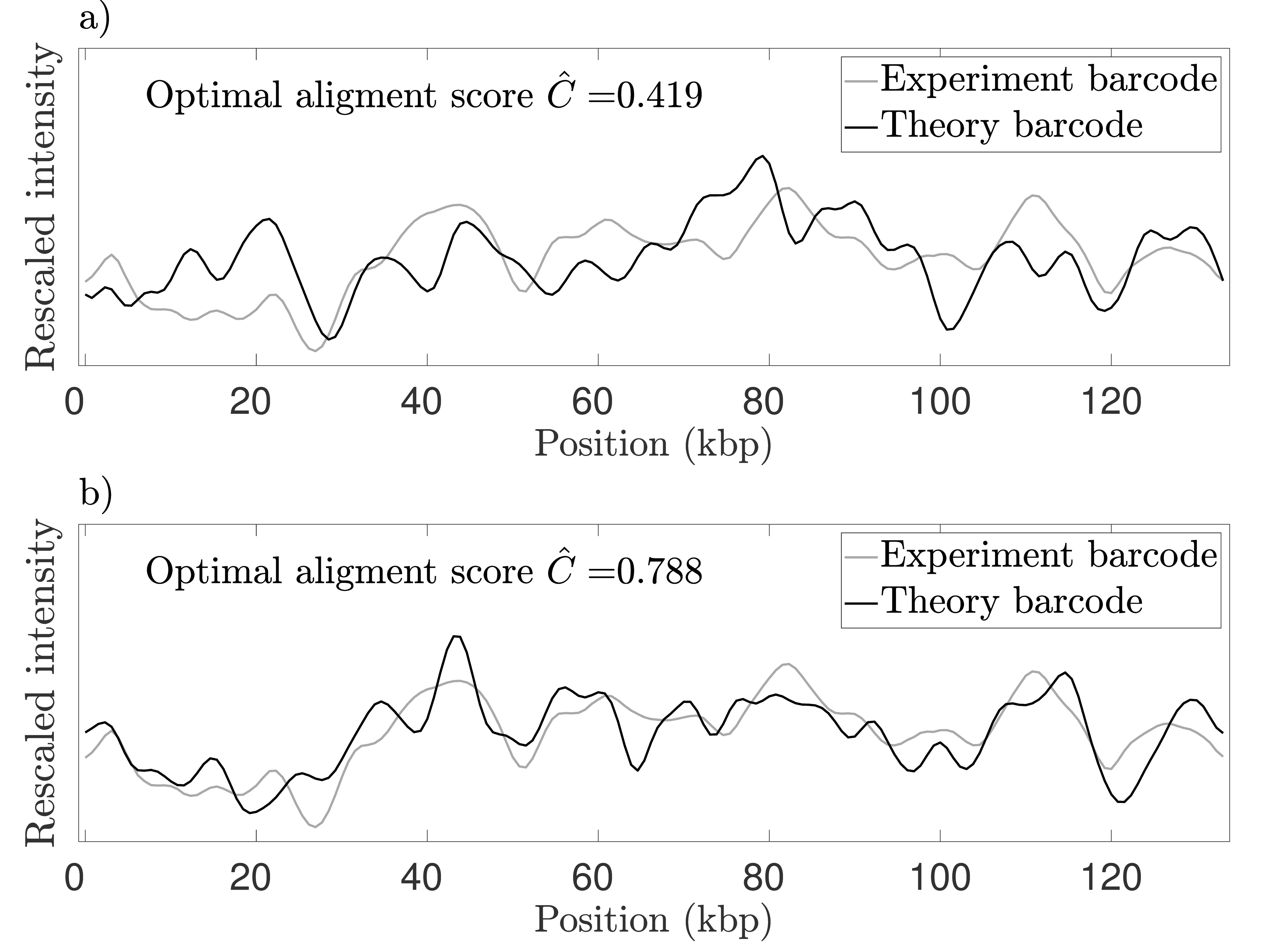

Supplement: S3 Fig — Two examples of randomized densely-labeled barcodes (competitive binding) compared to an experimental barcode of the same length. By generating many such randomized barcodes and matching experiments to these, we get optimal alignment score distributions of the form in Fig 2b) in the main text. (TIF) [file pone.0260489.s003.tif]

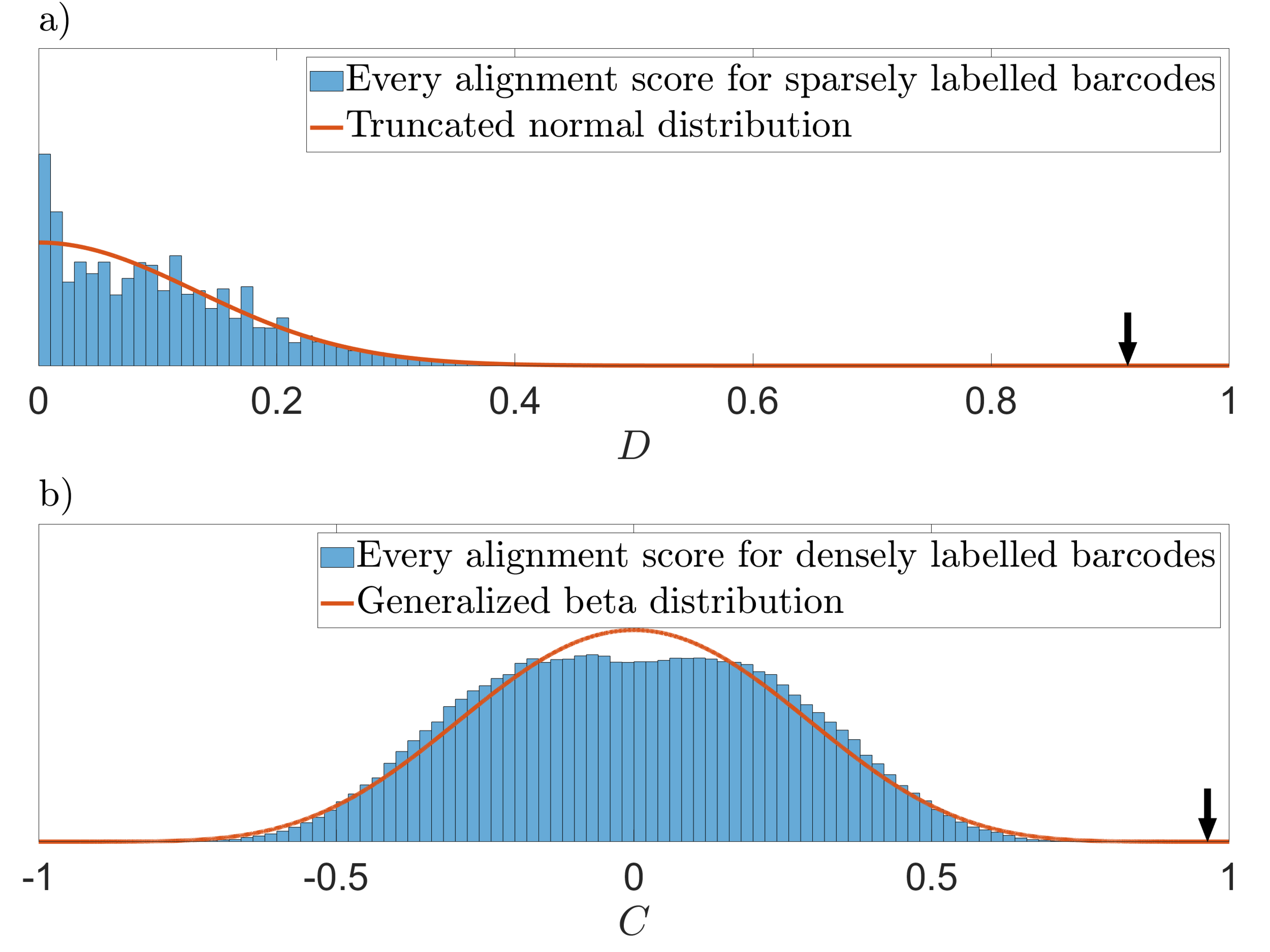

Supplement: S4 Fig — (a) When sparse-labeling alignment score, Eq (2) in the main text, is calculated between sufficiently long barcodes for every possible orientation and circular shift, the distribution is well described by a truncated normal distribution. (b) When the densely-labeling alignment score, Eq (3) in the main text, is calculated between sufficiently long barcodes for every possible orientation and circular shift, the distribution is centered around 0 and fitted by a functional form given in [27]. Compare these results to Fig 2 in the main text, which shows the associated optimal alignment score distribution for the best alignment. (TIF) [file pone.0260489.s004.tif]
