# Supplementary material for: Combining dense and sparse labeling in optical DNA mapping
Source: PLoS One. 2021 Nov 29;16(11):e0260489. doi: 10.1371/journal.pone.0260489 (PMC8629184; doi:10.1371/journal.pone.0260489)
Supplement: S1 File — Here we provide further details about the experiments and theoretical methods. (PDF) [file pone.0260489.s005.pdf]

Supporting Methods: Combining Dense and Sparse Labeling in Optical DNA Mapping

Erik Torstensson[1,◑] Gaurav Goyal[2,◑], Anna Johnning[3,4,5], Fredrik Westerlund[2,*], Tobias Ambjörnsson[1,*]

1 Department of Astronomy and Theoretical Physics, Lund University, Lund, Sweden
2 Department of Biology and Biological Engineering, Chalmers University of Technology, Gothenburg, Sweden
3 Department of Mathematical Sciences, Chalmers University of Technology and the University of Gothenburg, Gothenburg, Sweden
4 Systems and Data Analysis, Fraunhofer-Chalmers Centre, Gothenburg, Sweden
5 Centre for Antibiotic Resistance Research, CARe, University of Gothenburg, Gothenburg, Sweden
6 Department of Mathematical Sciences, Chalmers University of Technology/University of Gothenburg, Gothenburg, Sweden

◑These authors contributed equally to this work.
* tobias.ambjornsson@thep.lu.se (TA)

Experimental details

Here we provide further details about our experiments.

1 Bacterial strains and plasmids

The first plasmid considered in the main text is a 130 kbps long plasmid originally isolated from extended-spectrum beta-lactamase (ESBL)-producing *Escherichia coli* (*E. coli*). The plasmid was detected in multiple neonates during a nosocomial outbreak at Karolinska University Hospital in Stockholm, Sweden, between November 2008 and March 2009 [1, 2]. The plasmid used here was isolated from *E. coli* strain DA53965.

The second plasmid used in this study is a 220 kbps long plasmid originally isolated from a clinical *Klebsiella pneumoniae* (*K. pneumoniae*) strain that caused a hospital outbreak in Uppsala, Sweden [3]. The plasmid used here was isolated from *E. coli* strain DA24337.

2 Plasmid preparation

Escherichia coli cultures were grown overnight in low salt LB-medium (Sigma-Aldrich) at 37 °C. Plasmids were extracted using the NucleoBond Xtra Midi kit (Macherey-Nagel). For each isolate, 100 ml of the overnight culture was pelleted by centrifugation 5000 rcf, 10 minutes at 4 °C. The pellet was dissolved in resuspension buffer, lysed and purified on columns according to the manufacturer's recommendations. The eluted plasmid DNA was precipitated with isopropanol and washed once with 70% ethanol and dried at room temperature. The dried pellet was reconstituted in 100 μl TE-buffer and the DNA concentration was determined using Q-bit assay (Invitrogen).

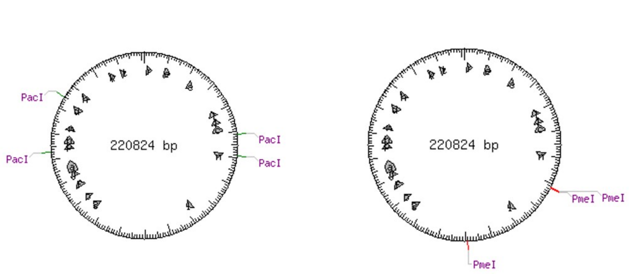

Fig 1. The 220 kbps plasmid theoretical digestion map. Expected digestion map for 220 kbps plasmid when digested with PacI, PmeI and SgrDI.

3 Plasmid digestion

NEB Cutter V2.0 (http://nc2.neb.com/NEBcutter2/) was used to perform *in silico* digestion of the two plasmids and identify restriction enzymes that could produce 3-5 cuts.

- 130 kbps plasmid (GenBank: CP025574.1): The first plasmid was digested with AscI enzyme (New England Biolabs) (3 cuts) or with PmeI enzyme (New England Biolabs) (5 cuts) at sub-optimal digestion conditions. AscI has 3 cut sites and PmeI has 5 cut sites on the plasmid. However, digestion conditions were optimized such that the plasmid is cut only at one of the cutting sites for each of the separate digestion reactions. To achieve sub-optimal digestion, enzyme concentration was titrated against the picomoles of cut sites in 0.5 μg plasmid DNA. For example, for AscI, the 130 kbps plasmid contains 3 cut sites or 0.0176 picomoles cut sites in 0.5 μg plasmid DNA. Since one unit activity of the AscI enzyme is defined as the enzyme needed to completely digest 0.0625 picomoles cut sites in lambda DNA (2 recognition sites) in one hour, we used 0.05 units of the enzyme for 30 minutes to partially digest 0.5 μg plasmid DNA. Restriction enzymes were freshly diluted in 1X Cut smart buffer (New England Biolabs) and the reaction was terminated after 30 minutes by heat inactivation at 70 °C for 20 minutes. Restriction enzyme resource from Promega was used for guidance [4].
- The 220 kbps plasmid (NCBI Reference Sequence: NC_016966.1): The plasmid was fully digested with SgrDI (ThermoFisher Scientific), PacI (New England Biolabs) or PmeI (New England Biolabs) in separate digestion reactions (digestion map of the 220 kbps plasmid for PacI, PmeI and SgrDI is shown in Fig. 1).

4 Plasmid staining

The digested plasmid samples were stained with YOYO-1 (YOYO, Invitrogen) and Netropsin (Sigma Aldrich) using ratio basepair: YOYO: netropsin :: 10: 1: 300. λ-DNA (48502 bps, New England Biolabs) was included in the sample as an internal size reference. First, samples were mixed in 0.5X TBE (Tris-Borate-EDTA, Medicago) and incubated at 50 °C for 30 minutes. Samples were then diluted with DNase free water to reach a final buffer concentration of 0.05X TBE and 0.2 μM (bp) DNA. Finally, 2% (v/v) β-mercaptoethanol (BME, Sigma-Aldrich) was included to prevent photo-nicking.

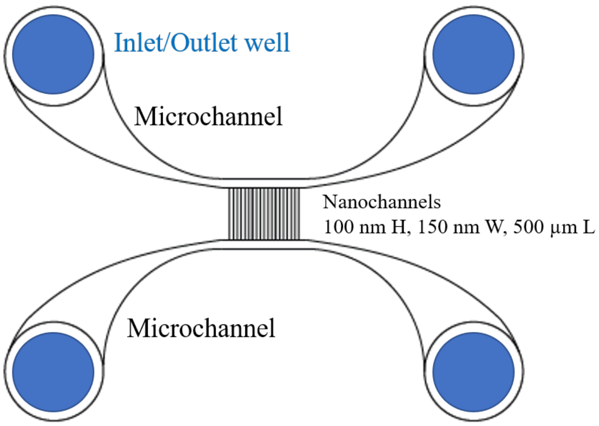

Fig 2. Schematic of the nanofluidic chip used for acquiring the experimental data. The chip consists of four inlet/outlet wells, where DNA and buffer solutions are loaded. The wells are connected by two microchannels which, in turn, are connected by nanochannels (height = 100 nm, width = 150 nm, length = 500 μm). Nitrogen gas was used to push DNA molecules from the wells into the microchannels and then into the nanochannels. Once the DNA molecules were confined in the nanochannels, the nitrogen pressure was turned off and DNA was imaged in no-flow condition. After imaging, the molecules were flushed out of the nanochannels, new molecules were pushed in and the process was repeated.

5 Nanochannel devices and DNA imaging

Nanofluidic chips were fabricated in fused silica at Chalmers University of Technology cleanroom facility using the process described elsewhere [5]. The chip consisted of two inlet wells connected by a microchannel and two outlet wells connected by a microchannel, see Fig. 2. The two microchannels were connected by an array of nanochannels of dimensions of 100 $\times$ 150 nm^2, and 500 μm long. To achieve uniform conditions, the channels were pre-wetted with 0.05X TBE buffer and 2% v/v BME. A sample volume of 10 μL was loaded into one of the inlet wells and DNA molecules were forced into the nanofluidic channels using a pressure-driven flow of nitrogen gas. When in nanochannels, the DNA molecules stretch to 70% - 90% of their contour length, and the amount of stretching is calculated by imaging size reference λ-DNA.

Once stretched, the stained DNA molecules were imaged using an inverted microscope (Zeiss AxioObserver.Z1) with a 100X oil immersion objective (Zeiss, NA = 1.46) and an EMCCD camera (Photometrix Evolve). In total, a series of up to 50 images with an exposure time of 100 ms were obtained from each DNA molecule to obtain the sequence-specific barcode.

A minimal enzyme library

The enzyme assay is strictly dependent on the enzymes used to cut the plasmids. It is therefore important to select the best enzymes to use, which can be tricky for a plasmid sample with unknown content. The aim was to find the library of restrictions enzymes where the highest proportion of plasmids would be cleaved suitably by at least one of the enzymes. An enzyme was considered to cut a plasmid suitably if it had at least two restriction sites and all sites were at least 10 kbps apart. To identify this library, EMBOSS (v. 6.5.7.0) [6] restrict was applied to the circular plasmid sequences using all commercially available enzymes in the REBASE database [7] with at least 4

bp long non-methylated recognition sites (parameters –enzymes all –sitelen 4 –plasmid Y –commercial –methylation N). All plasmid sequences were retrieved from the NCBI RefSeq database (2021-05-18). We then made a further refinement of the RefSeq database, as described in the Methods section in the main text. From the results, the proportion of reference plasmids cut suitably by at least one member of the enzyme library was calculated for all possible combinations of up to five enzymes. This analysis makes it possible to select the best enzymes for the study at hand. The number of enzymes selected can be varied based on the resources at hand. If only one single experiment is possible the enzyme AvrII cuts 18.0 % of the plasmids in the preferred fashion. Increasing the number of enzymes to five leads to a library of enzymes that covered 862 (60.7 %) of the plasmids: AvrII, FspAI, PmeI, SgfI, and SpeI. If the same library of enzymes was applied only to reference plasmid with an Enterobacterales family/genus/species in the fasta header, 342 out of 455 (75.2 %) plasmids would be cut suitably by at least one of the enzymes. For other experiments the enzyme library can be adjusted based on the sample of interest and the resources at hand.

Consensus of cut-labeled barcodes and false cut rejection

Our experimental sequence-specific cut-labeling assay is described above and in the Methods section in the main text. Due to, for instance, photo-induced nicking events, a cut may occur at a non-specific site – we here refer to such cuts as 'false cuts'. In order to discard false cuts, and to refine the measured locations of "real" enzymatic cuts, we use a Monte-Carlo simulation that we refer to as the extended balls-in-boxes method. The new method is similar to the method presented in [8], but extended to handle more than one cut per molecule.

The purpose of the simulation is to provide a null model from which we can estimate the means and standard deviations of the number of cuts that could be found in a small region of the DNA barcode if the cuts occur at random locations (uniformly distributed on the DNA). To that end, in our extended balls-in-boxes method, we randomly throw imagined 'balls' (cuts) into 'boxes' (pixels). In order to deal with the limited spatial resolution in the experiment, we then merge consecutive boxes into bins (clusters) of chosen width and overlapping position. We then estimate the expected number of balls $\mu_{\text{balls},1}$ in the bin with the most number of balls ("1" refers to the "best case", i.e., the most-filled bin) and the associated standard deviation $\sigma_{\text{balls},1}$. With a null model at hand, we then turn to the actual experimental data and count the number of cuts in the most-filled bin. A bin (cluster of cuts) is considered statistically significant if it contains a number of experimental cuts which is 3 $\sigma_{\text{balls},1}$ above the estimated mean, $\mu_{\text{balls},1}$. If the most-filled bin count is deemed significant, we go on to consider the bin which had the second most number of cuts (which did not overlap with the most-filled bin), and investigate whether this bin had a significant number of cuts, etc.

In detail, our extended balls-in-boxes method is:

1. Set a counter for the number of significant clusters to $i = 1$.
2. Denote by N_p the DNA barcode length in pixels, and by N the number of observed cuts in the experiment.
3. Calculate the number of cuts in the N_p (overlapping) bins of width W.
4. Generate a null model. To that end, repeat 1000 times:

(a) Randomly place N balls in N_p boxes (uniform distribution).
(b) Calculate the number of balls in each bin of width W.
(c) Make a note of the number of balls in the bin with the most number of balls.

5. Use the output from step 3. to estimate the mean $\mu_{\text{balls},i}$ and standard deviation $\sigma_{\text{balls},i}$ of the number of simulated balls in the bins with the most number of balls.

6. If the number of experimental cuts in the bin with the most number of experimental cuts is larger than 3 $\sigma_{\text{balls},i}$ above the mean $\mu_{\text{balls,i}}$, consider it a statistically significant cluster of experimental cuts. Calculate the mean position of experimental cuts in the bin and use that as a sequence-specific marker.

7. Remove clustered experimental cuts from the pool of experimental cuts (and update N accordingly), exclude the pixel locations covered by the cluster (and update N_p accordingly), change $i \to i+1$ and repeat from step 2. until there are no more significant clusters.

In our null model simulations, the bin width is chosen to be $W = 6$ pixels. This width was chosen to be comparable to the theoretical width of a Gaussian distribution with standard deviation σ_{PSF} that has a full width at half maximum (FWHM) $\approx 2.36\sigma_{PSF} \approx 4.4$ pixels. We choose the bin width to be somewhat larger than this value to encompass another source of error that contributes to the expected bin width, namely the inherent uncertainty in the detection of DNA-fragment ends.

Derivation of the expression for $\mathcal{N}^{\mathbf{EV}}_{\mathbf{PDF}}\,(x|\mu,\sigma,\lambda)$

In order to convert our alignment score for sparsely-labeled DNA barcodes (see the Methods section in the main text) into p-values, we seek the probability density function (PDF), $w(\hat{D})$, of the best score ($\hat{D}$) out of a set of random alignment scores. We find the solution to this problem in extreme-value statistics [9, 10]. Let us briefly recapitulate the main results from this field, adapted to the present problem. Denote by $f(D)$ the PDF for the null model alignment scores and by $F(D)$ the associated cumulative distribution function (CDF). We are then interested in the distribution of the best score, $\hat{D}$, given a set of n scores. Let us denote by $W(\hat{D})$ the CDF associated with $w(\hat{D})$. The quantity $W(\hat{D})$ is simply the probability that all n scores are less than $\hat{D}$, and is hence given by

$$W(\hat{D}) = [F(\hat{D})]^n \tag{1}$$

The PDF $w(\hat{D})$ is then found from the derivative of the CDF:

$$w(\hat{D}) = \frac{\partial}{\partial \hat{D}}[F(\hat{D})]^n = n[F(\hat{D})]^{n-1}\frac{\partial F(\hat{D})}{\partial \hat{D}}\,. \tag{2}$$

The results above are general and only assume that the n random scores are independent and identically distributed.

In our case, for sparse labeling, we have an alignment score (D) given in Eq (2) in the main text. We now seek an approximate expression for the distribution of this score. We notice two main features of our choice of score: (i) it consists of two sums; (ii) it is bounded to the domain $[0, 1]$. These two features lead us to propose the following functional form

$$f(D) = f(D|\mu,\sigma,\lambda) = \frac{1}{\sigma}\frac{\mathcal{N}_{\text{PDF}}\,((D-\mu)/\sigma)}{\mathcal{N}_{\text{CDF}}\,((1-\mu)/\sigma) - \mathcal{N}_{\text{CDF}}\,(-\mu/\sigma)}, \tag{3}$$

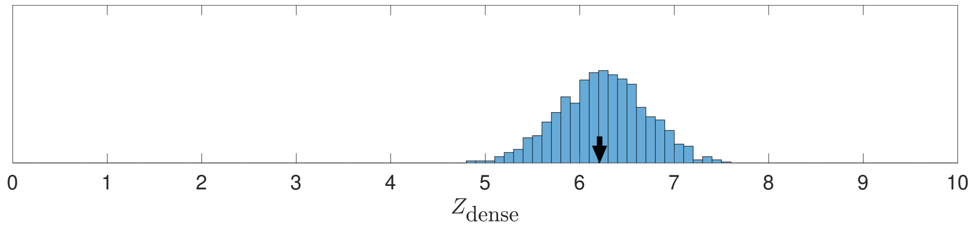

Fig 3. Validation of synthetic barcodes. We generated synthetic competitive binding barcodes through the procedure described in Methods in the main text (130 kbps plasmid). This procedure was repeated 1000 times, and the resulting match scores, Z_{dense}, were obtained and turned into histograms. Our procedure for calculating match scores is described in the Methods section in the main text. The arrow in each panel represents match scores of the real experiment against their "true" theoretical barcode. Notice that our procedure for generating synthetic barcodes have a mean close to the mean score for actual experiments.

where $\mathcal{N}_{\text{CDF}}(y) = \left(1 + \text{erf}\left(y/\sqrt{2}\right)\right)/2$ and $\mathcal{N}_{\text{PDF}}(y) = \exp\left(-y^2/2\right)/\sqrt{2\pi}$. The assumption of a normal distribution is based on point (i) above together with the central limit theorem (i.e., we assume that we have "many" dots). We truncate the normal distribution at $D = 0$ and $D = 1$ in order to conform with the property (ii) of our choice of score.

By inserting $f(D)$ as given in Eq (3) into Eqs. (1) and (2), we arrive at Eqs. (4) and (6) in the main text. Note that the parameters in the distribution for $\hat{D}$ must be interpreted as effective parameters due to the correlation between pixels caused by the optical point spread function (compare to [11]).

Validation of our method for generating synthetic CB barcodes.

We here validate our procedure for generating synthetic CB barcodes, as described in the Methods section in the main text. To that end, we generated 1000 synthetic barcodes (noisified theoretical barcodes) from the DNA sequence for 130 kbps plasmid (see main text). We then matched these 1000 synthetic barcodes against the correct theoretical barcode. The match scores from synthetic barcode matchings are then compared to the match scores of the real experiment (matched against the correct theoretical barcode) in Figure 3. We find that indeed our synthetic barcodes give match scores which, on average, are very close the those of experiments.

References

1. Nordberg V, Jonsson K, Giske CG, Iversen A, Aspevall O, Jonsson B, et al. Neonatal intestinal colonization with extended-spectrum β-lactamase–producing Enterobacteriaceae—a 5-year follow-up study. Clinical Microbiology and Infection. 2018;24(9):1004 – 1009.

2. Bikkarolla S, Nordberg V, Rajer F, Müller V, Kabir M, K K S, et al. Optical DNA Mapping Combined with Cas9-Targeted Resistance Gene Identification for Rapid Tracking of Resistance Plasmids in a Neonatal Intensive Care Unit Outbreak. mBio. 2019;10(4):e00347–19.

3. Sandegren L, Linkevicius M, Lytsy B, Melhus Å, Andersson D. Transfer of an Escherichia coli ST131 multiresistance cassette has created a Klebsiella pneumoniae-specific plasmid associated with a major nosocomial outbreak. The Journal of antimicrobial chemotherapy. 2012;67:74–83.
4. Promega. Restriction Enzyme Resource - Restriction Enzyme Activity;. Available from: `https://se.promega.com/resources/guides/nucleic-acid-analysis/restriction-enzyme-resource/#rest-activity`.
5. Persson F, Tegenfeldt J. DNA in nanochannels-directly visualizing genomic information. 2010;39(3):985–999.
6. Rice P, Longden I, Bleasby A. EMBOSS: the European molecular biology open software suite. Trends in genetics. 2000;16(6):276–277.
7. Roberts RJ, Vincze T, Posfai J, Macelis D. REBASE—a database for DNA restriction and modification: enzymes, genes and genomes. Nucleic acids research. 2015;43(D1):D298–D299.
8. Müller V, Rajer F, Frykholm K, K Nyberg L, Quaderi S, Fritzsche J, et al. Direct identification of antibiotic resistance genes on single plasmid molecules using CRISPR/Cas9 in combination with optical DNA mapping. Scientific Reports. 2016;6(1):37938.
9. Gumbel EJ. Statistics of Extremes, Columbia Univ. Press, New York. 1958; p. 201.
10. Schmittmann B, Zia RKP. "Weather" records: Musings on cold days after a long hot Indian summer. American Journal of Physics. 1999;67(12):1269–1276.
11. Dvirnas A, Pichler C, Stewart C, Quaderi S, K Nyberg L, Müller V, et al. Facilitated sequence assembly using densely labeled optical DNA barcodes: A combinatorial auction approach. PLOS One. 2018;13(3):e0193900.
